# Supplementary material for: Paradoxical non-catalytic kinase functions are driven by inhibitor-induced displacement of autoinhibitory domains
Source: Mol Syst Biol. 2026 Jul 2;22(9):1474–500. doi: 10.1038/s44320-026-00229-2 (PMC13538393; doi:10.1038/s44320-026-00229-2)
Supplement: Supplementary file 1 [file 44320_2026_229_MOESM14_ESM.pdf]

Expanded View Figures

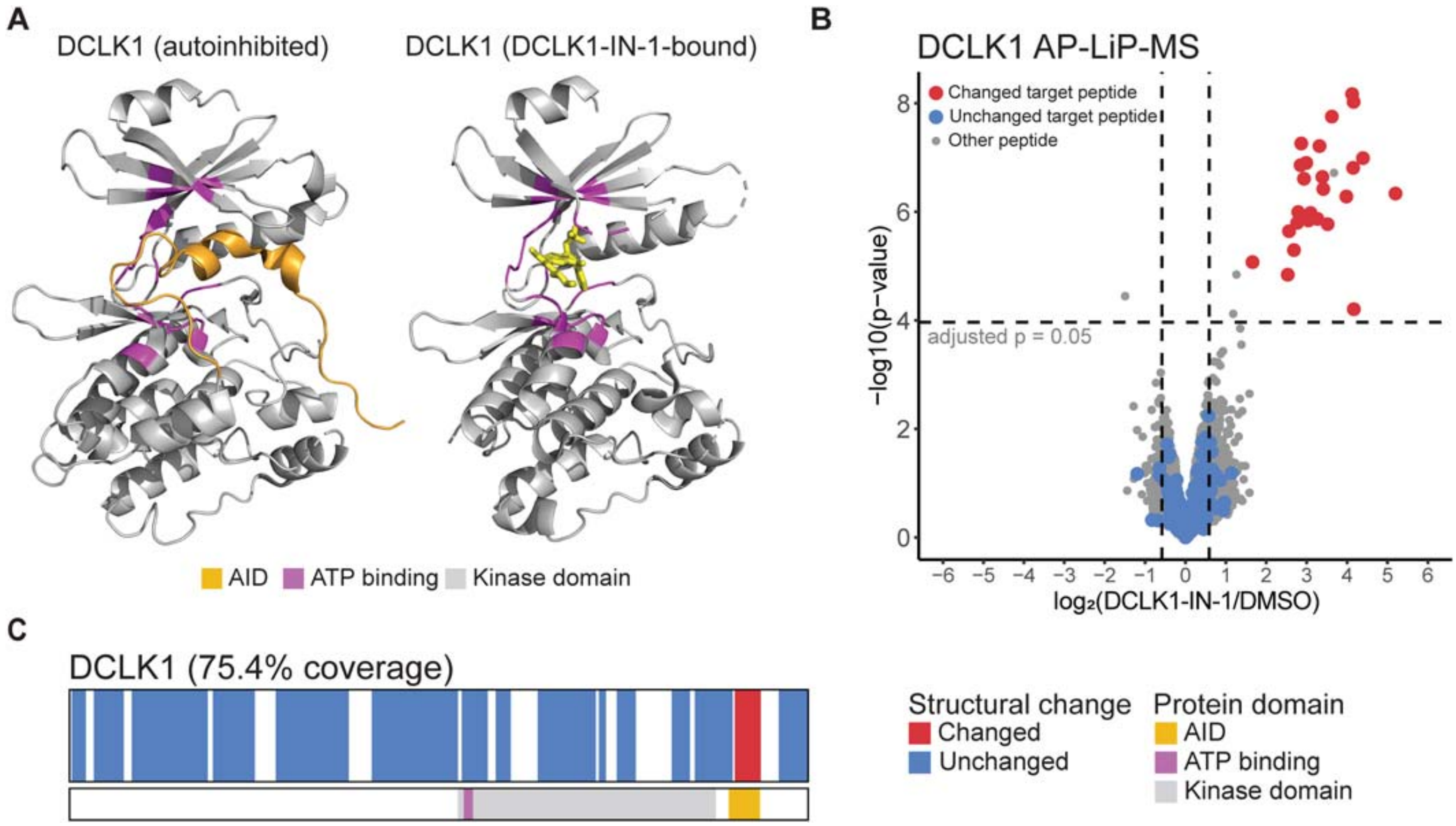

Figure EV1. Validation of AP-LiP-MS using DCLK1 inhibited with DCLK1-IN-1.

(**A**) Comparison of the resolved structures of DCLK1 in the autoinhibited configuration (PDB: 6KYQ) and the DCLK1-IN-1-bound configuration (PDB: 7KXW; the inhibitor DCLK1-IN-1 is shown in yellow). (**B**) Volcano plot showing LiP peptide intensity changes in the DCLK1-enriched samples upon treatment with DCLK1-IN-1 compared to vehicle control. (**C**) Barcode plot of structural changes in DCLK1 in response to inhibition with DCLK1-IN-1. The AP-LiP-MS experiment was performed in technical triplicates. *P* values were calculated using a moderated *t* test followed by multiple testing corrections using the procedure of Benjamini–Hochberg. Source data are available online for this figure.

© The Author(s)

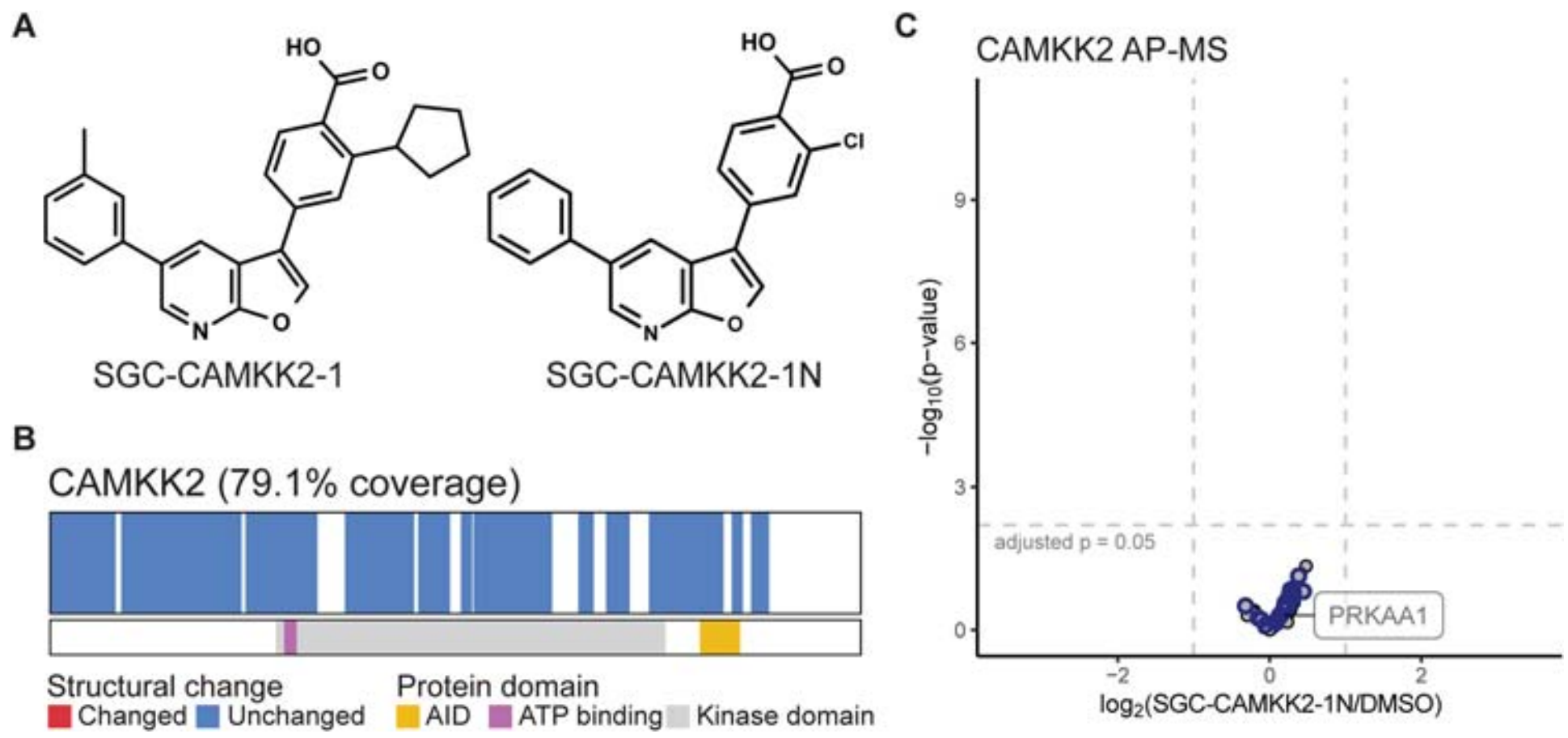

Figure EV2. The negative control compound SGC-CAMKK2-1N does not induce structural or interaction changes in CAMKK2.

(**A**) Structures of the inhibitor SGC-CAMKK2-1 and the negative control compound SGC-CAMKK2-1N. (**B**) Structural changes observed with AP-LiP-MS for CAMKK2 treated with SGC-CAMKK2-1N. (**C**) Volcano plot illustrating changes in CAMKK2 interactors upon inhibition with SGC-CAMKK2-1N measured by AP-MS. Known CAMKK2 interactors are highlighted with a blue border. AP-LiP-MS experiments were performed in technical triplicates, and AP-MS experiments were performed in biological triplicates. *P* values were calculated using a moderated *t* test followed by multiple testing corrections using the procedure of Benjamini–Hochberg. Source data are available online for this figure.

 © The Author(s)

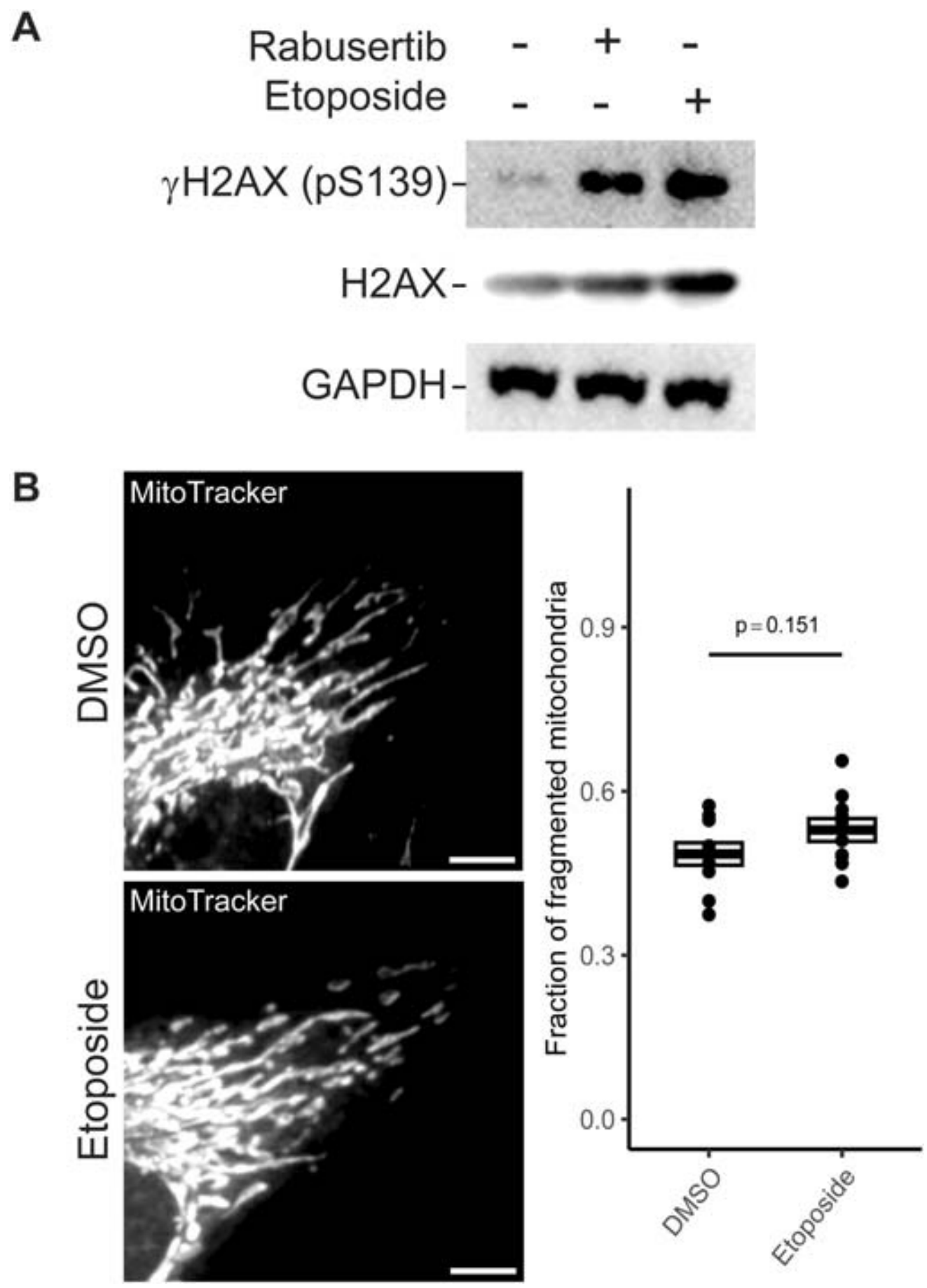

Figure EV3. DNA damage is induced by rabusertib treatment but does not underlie mitochondrial fragmentation.

(**A**) Western blot analysis of DNA damage in HEK cells upon treatment with rabusertib (4 h) or etoposide (3 h). (**B**) Mitochondria of HeLa cells treated with etoposide. Data points are summarized as Tukey box plots where the center line denotes the median, and the lower and upper bounds represent the 25th and 75th percentiles, respectively. The *P* value was calculated using a two-tailed *t* test. For each condition, ten images taken across three biological replicates were quantified. Source data are available online for this figure.

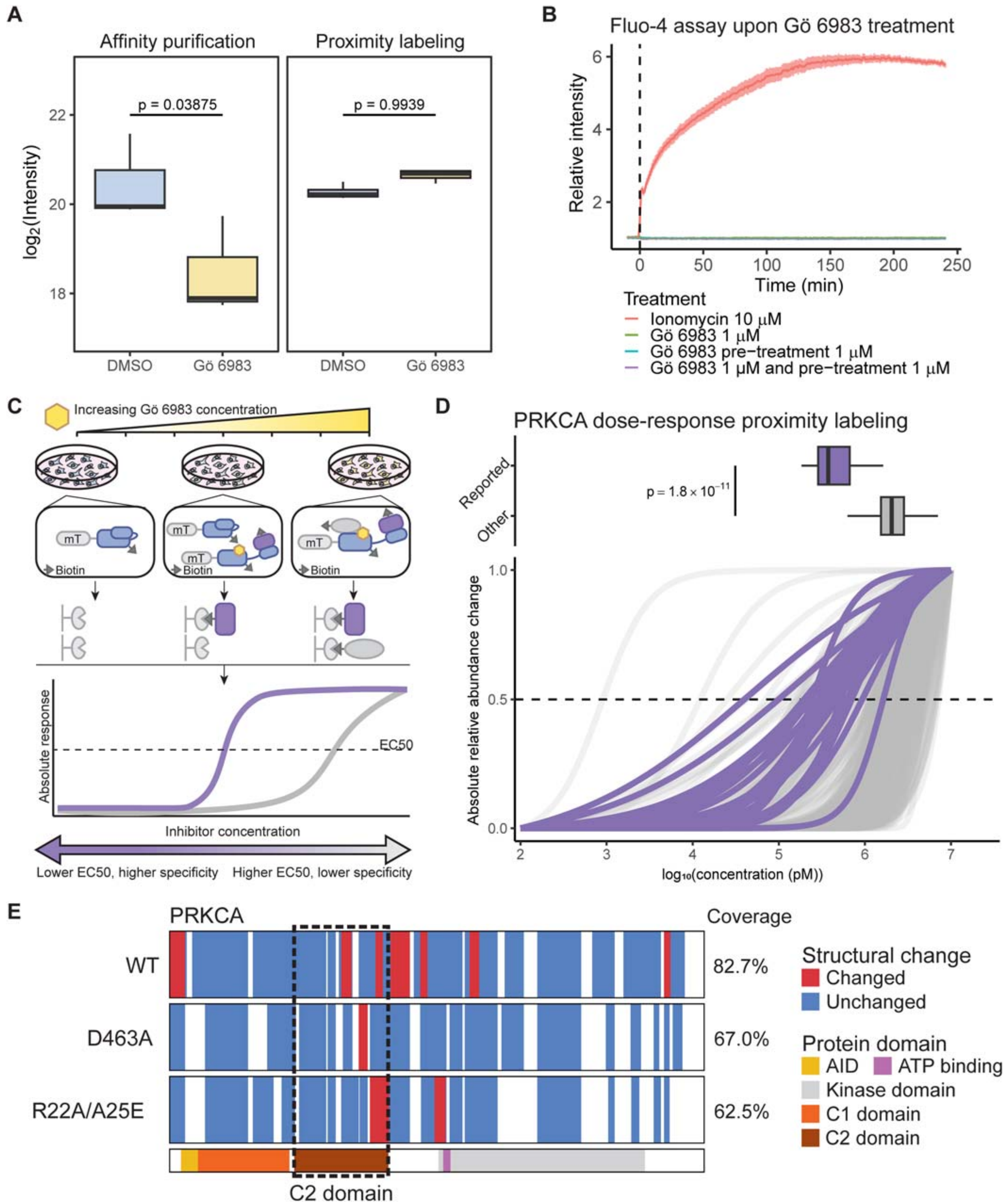
A
Affinity purification
Proximity labeling
p = 0.03875
p = 0.9939
log2(Intensity)
DMSO
Gö 6983
DMSO
Gö 6983
B
Fluo-4 assay upon Gö 6983 treatment
Relative intensity
Time (min)
Treatment
Ionomycin 10 μM
Gö 6983 1 μM
Gö 6983 pre−treatment 1 μM
Gö 6983 1 μM and pre−treatment 1 μM
C
Increasing Gö 6983 concentration
mT
Biotin
Absolute response
EC50
Inhibitor concentration
Lower EC50, higher specificity
Higher EC50, lower specificity
D
PRKCA dose-response proximity labeling
Reported
Other
p = 1.8 × 10−11
Absolute relative abundance change
log10(concentration (pM))
E
PRKCA
Coverage
WT
82.7%
D463A
67.0%
R22A/A25E
62.5%
Structural change
Changed
Unchanged
Protein domain
AID
ATP binding
Kinase domain
C1 domain
C2 domain
C2 domain

Figure EV4. Gö 6983-induced PRKCA membrane relocalization correlates with structural changes in the C2 domain.

(**A**) Differences in PRKCA abundance upon treatment with Gö 6983 in affinity purification and proximity labeling experiments. AP-MS was performed in biological triplicates, and proximity labeling was performed in biological quadruplicates. Data points are summarized as Tukey box plots where the center line denotes the median, and the lower and upper bounds represent the 25th and 75th percentiles, respectively. The whiskers represent the lowest or highest data point that is less than 1.5-fold the interquartile range from the box edges. The *P* values were calculated using a one-tailed *t* test. (**B**) Changes in intracellular Ca^{2+} concentration in response to Gö 6983 or ionomycin as a positive control measured using a Fluo-4 calcium imaging assay. For each time point, the mean and the standard error of the mean are reported. For each condition, six technical replicates were used. (**C**) Schematic representation of the dose–response in vivo proximity labeling workflow. (**D**) Dose–response curves for proteins changing with increasing Gö 6983 concentrations. Proteins reported to change in Fig. 6A are shown in purple; all other proteins are shown in gray. For each dose, biological quadruplicates were used. Data points are summarized as Tukey box plots where the center line denotes the median, and the lower and upper bounds represent the 25th and 75th percentiles, respectively. The whiskers represent the lowest or highest data point that is less than 1.5-fold the interquartile range from the box edges. The *P* value comparing EC50s was calculated using a two-sided Mann–Whitney *U* test. (**E**) Gö 6983-induced structural changes obtained by AP-LiP-MS in wild-type and mutant PRKCA. The panel for PRKCA WT was previously shown in Fig. 1D. AP-LiP-MS experiments were performed in technical triplicates. *P* values were calculated using a moderated *t* test followed by multiple testing corrections using the procedure of Benjamini–Hochberg. Source data are available online for this figure.
