# Supplementary material for: Paradoxical non-catalytic kinase functions are driven by inhibitor-induced displacement of autoinhibitory domains
Source: Mol Syst Biol. 2026 Jul 2;22(9):1474–500. doi: 10.1038/s44320-026-00229-2 (PMC13538393; doi:10.1038/s44320-026-00229-2)
Supplement: Supplementary file 2 [file 44320_2026_229_MOESM1_ESM.pdf]

# Appendix for “Paradoxical non-catalytic kinase functions are driven by inhibitor-induced displacement of autoinhibitory domains”

**Table of contents**

Figures:

Appendix Figure S1 page 2
Appendix Figure S2 page 3
Appendix Figure S3 page 4
Appendix Figure S4 page 5
Appendix Figure S5 page 6
Appendix Figure S6 page 7
Appendix Figure S7 page 8
Appendix Figure S8 page 9
Appendix Figure S9 page 10
Appendix Figure S10 page 11
Appendix Figure S11 page 12

Tables:

Appendix Table S1 page 13
Appendix Table S2 page 14
Appendix Table S3 page 16
Appendix Table S4 page 17
Appendix Table S5 page 19
Appendix Table S6 page 20
Appendix Table S7 page 21
Appendix Table S8 page 22
Appendix Table S9 page 23
Appendix Table S10 page 24

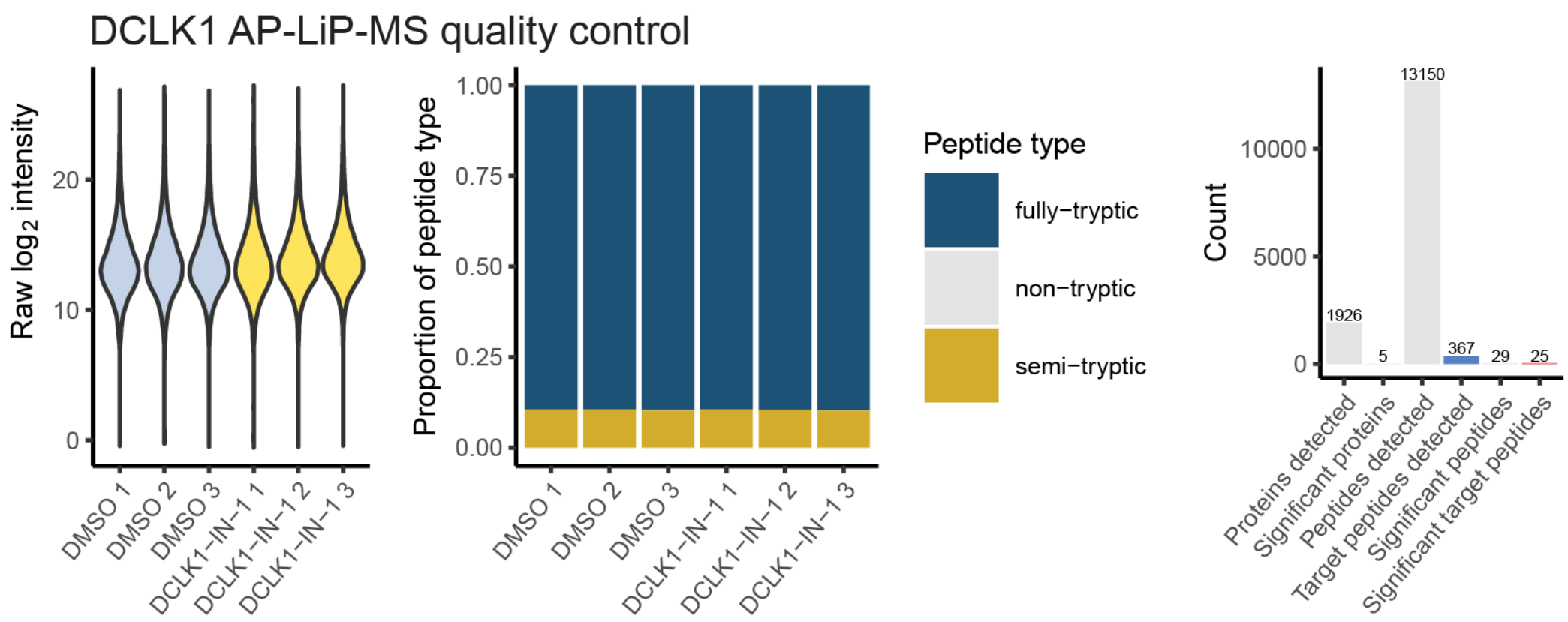


**Appendix Figure S1. Quality control for AP-LiP-MS data relating to Fig. EV1.** Raw intensities, raw peptide type distribution, and summary of metrics related to the peptides quantified for DCLK1. AP-LiP-MS experiments were performed in technical triplicates and p-values were calculated using a moderated t-test followed by multiple testing corrections using the procedure of Benjamini-Hochberg.

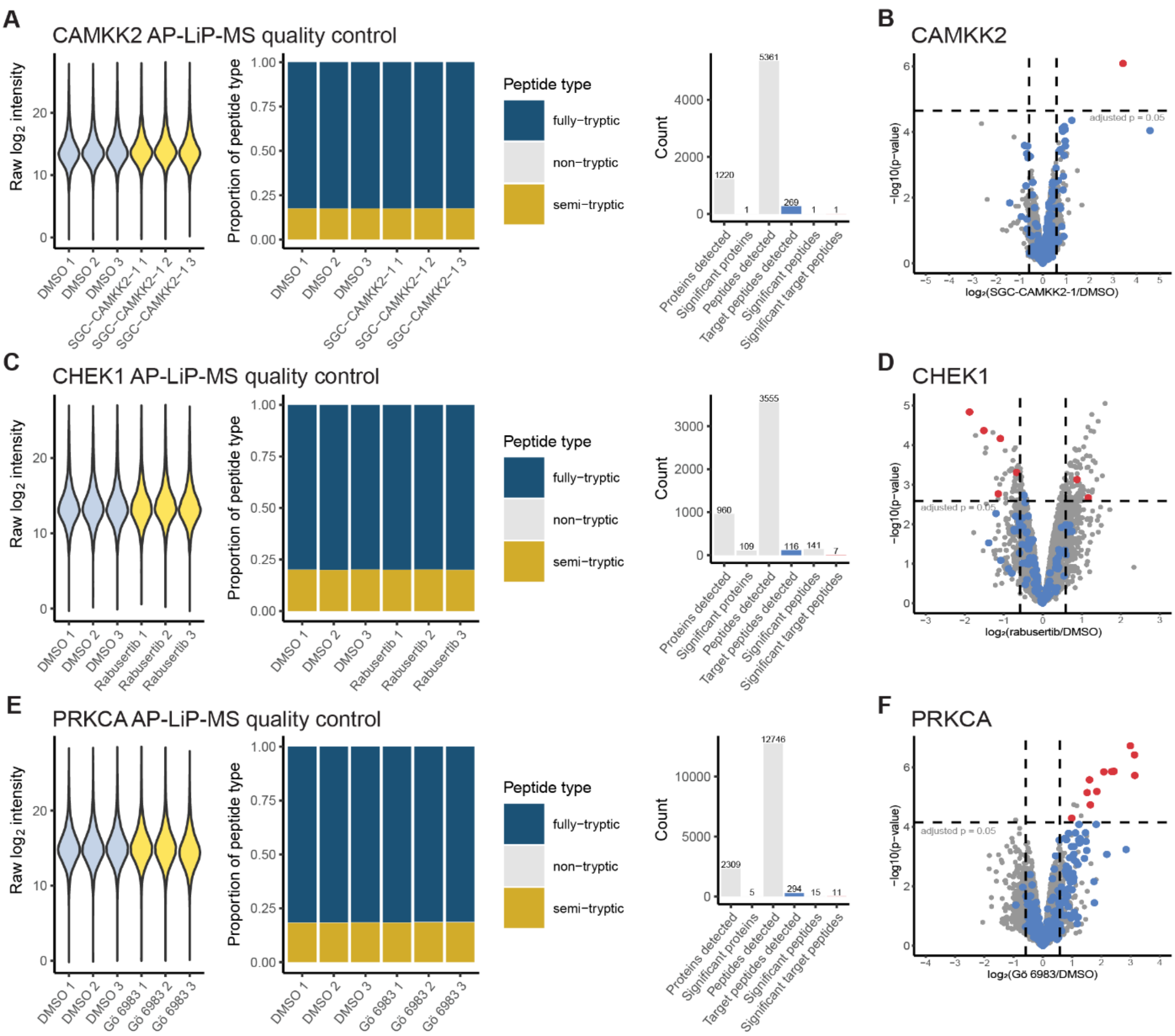


**Appendix Figure S2. Quality control for AP-LiP-MS data relating to Fig. 1.** A, C, E) Raw intensities, raw peptide type distribution, and summary of metrics related to the peptides quantified for CAMKK2, CHEK1 and PRKCA, respectively. B, D, F) Volcano plot showing LiP peptide intensity changes in the kinase-enriched samples for CAMKK2, CHEK1, and PRKCA, respectively, upon treatment with SGC-CAMKK2-1, rabusertib, or Gö 6983, compared to vehicle control. In red are significantly changing target peptides, in blue are the non-changing target peptides. Peptides from other proteins are shown in grey. All AP-LiP-MS experiments were performed in technical triplicates and p-values were calculated using a moderated t-test followed by multiple testing corrections using the procedure of Benjamini-Hochberg.

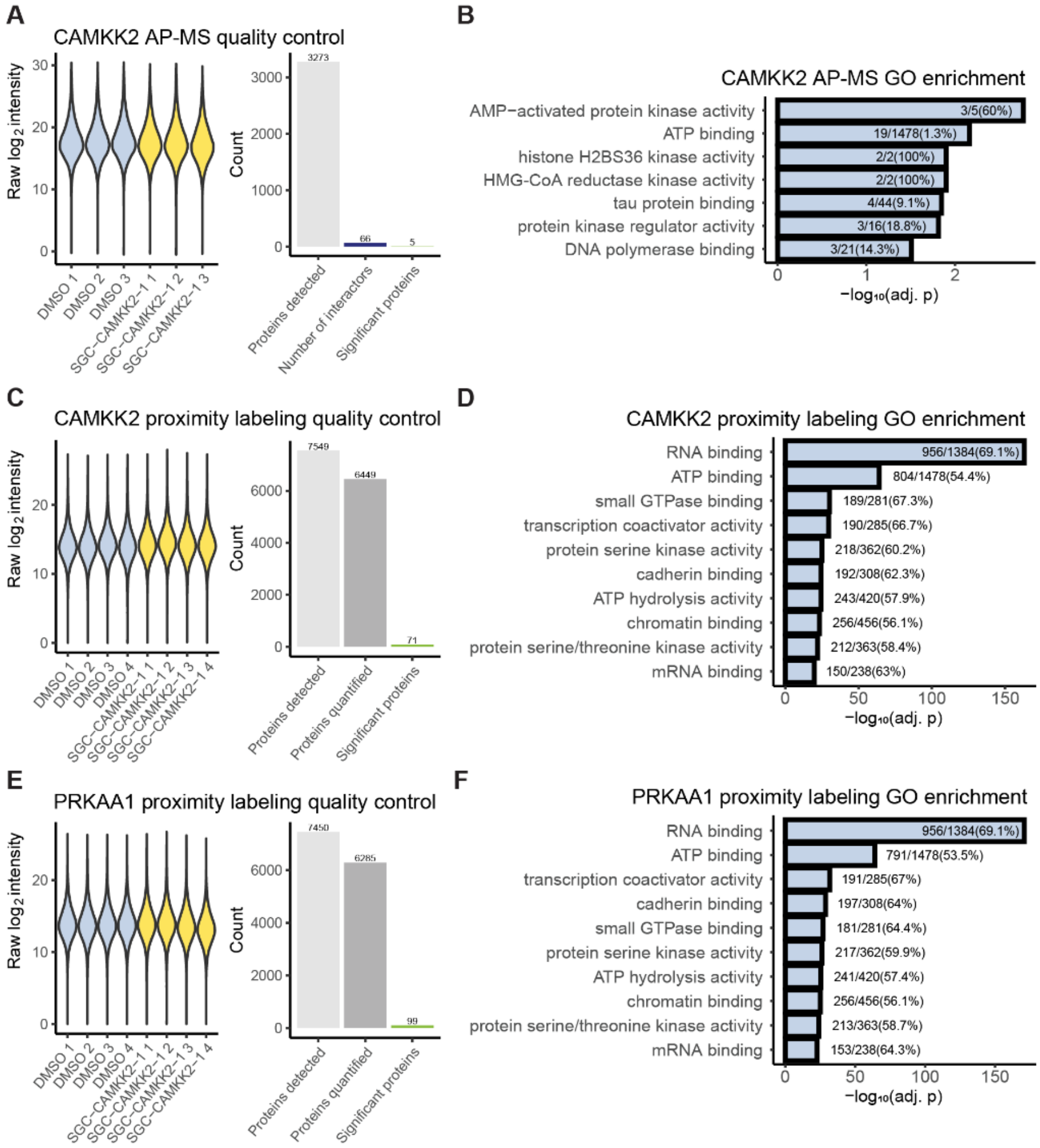


**Appendix Figure S3. Quality control for characterization of CAMKK2 complexes and proximity upon SGC-CAMKK2-1 treatment relating to Fig. 2.** A, C, E) Raw intensities and summary of metrics related to the proteins quantified for CAMKK2 AP-MS, CAMKK2 *in vivo* proximity labeling, and PRKAA1 *in vivo* proximity labeling, respectively. B, D, F) GO enrichment analysis (molecular function) across all CAMKK2 interactors identified in the AP-MS experiment, or all proteins quantified in the *in vivo* proximity labeling experiment using CAMKK2 or PRKAA1 as bait, respectively. AP-MS experiments were performed in biological triplicates, and *in vivo* proximity labeling experiments in biological quadruplicates. P-values were calculated using a Fisher's exact test followed by multiple testing corrections using the procedure of Benjamini-Hochberg.

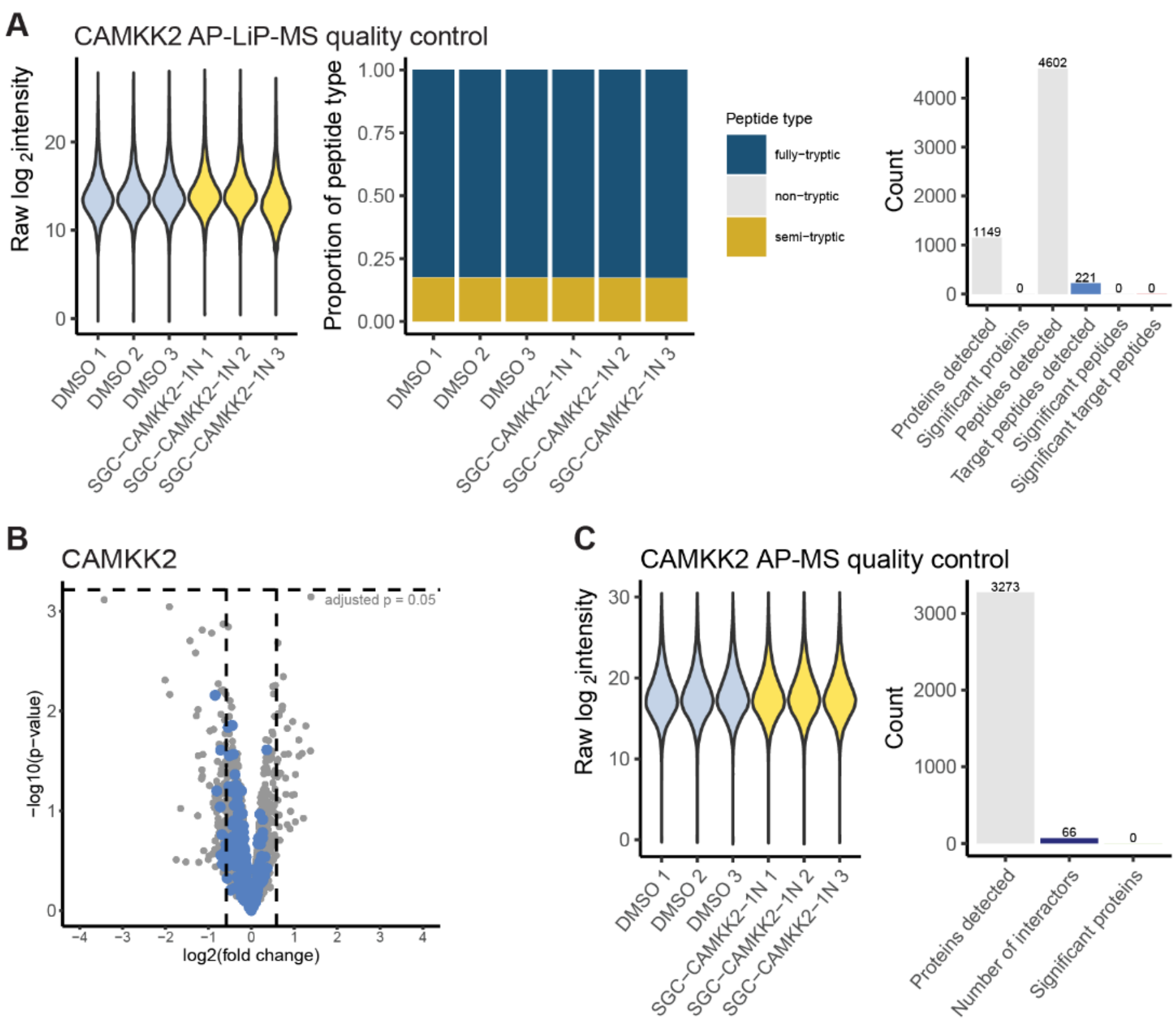


**Appendix Figure S4. Quality control for characterization of effects of SGC-CAMKK2-1N on CAMKK2 structure and complexes Fig. EV2.** A) Raw intensities, raw peptide type distribution, and summary of metrics related to the peptides quantified for CAMKK2. B) Volcano plot showing LiP peptide intensity changes in the kinase-enriched samples for CAMKK2 upon treatment with SGC-CAMKK2-1N compared to vehicle control. In red are significantly changing target peptides, in blue are the non-changing target peptides. Peptides from other proteins are shown in grey. C) Raw intensities and summary of metrics related to the proteins quantified for CAMKK2 AP-MS. AP-LiP-MS experiments were performed in technical triplicates and AP-MS experiments were performed in biological triplicates and p-values were calculated using a moderated t-test followed by multiple testing corrections using the procedure of Benjamini-Hochberg.

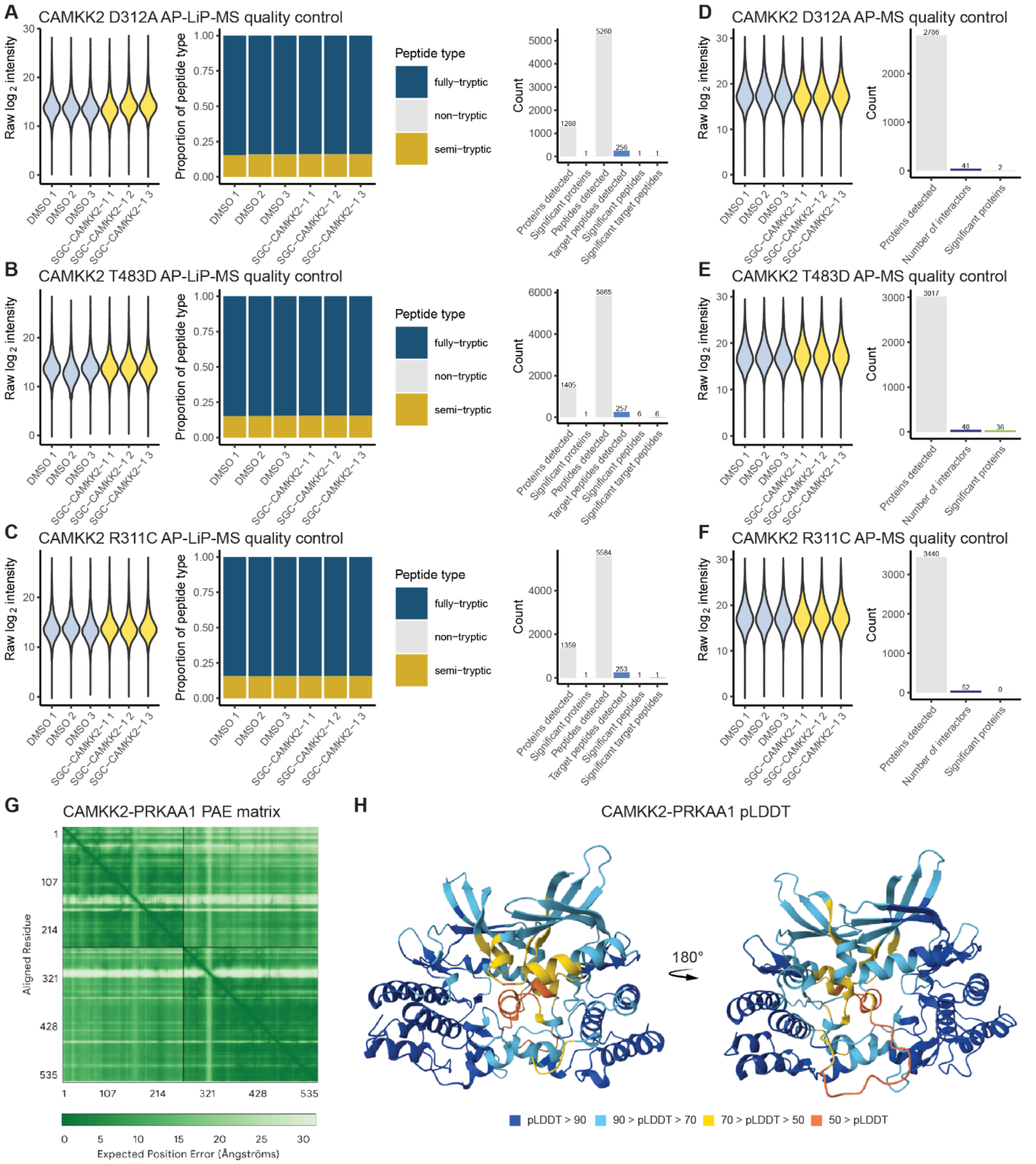


**Appendix Figure S5. Quality control for CAMKK2-PRKAA1 complex characterization relating to Fig. 3.** A, B, C) Raw intensities, raw peptide type distribution, and summary of metrics related to the peptides quantified for AP-LiP-MS to analyze changes in CAMKK2 D312A upon SGC-CAMKK2-1 treatment. D, E, F) Raw intensities and summary of metrics related to the proteins quantified for CAMKK2 D312A, T483D and R331C AP-MS, respectively. G) Predicted Aligned Error (PAE) matrix for the CAMKK2-PRKAA1 AlphaFold3 prediction. H) The predicted local distance difference test (pLDDT) score mapped onto the predicted structure of the CAMKK2-PRKAA1 structure from two different views. AP-LiP-MS experiments were performed in technical triplicates and AP-MS experiments were performed in biological triplicates.

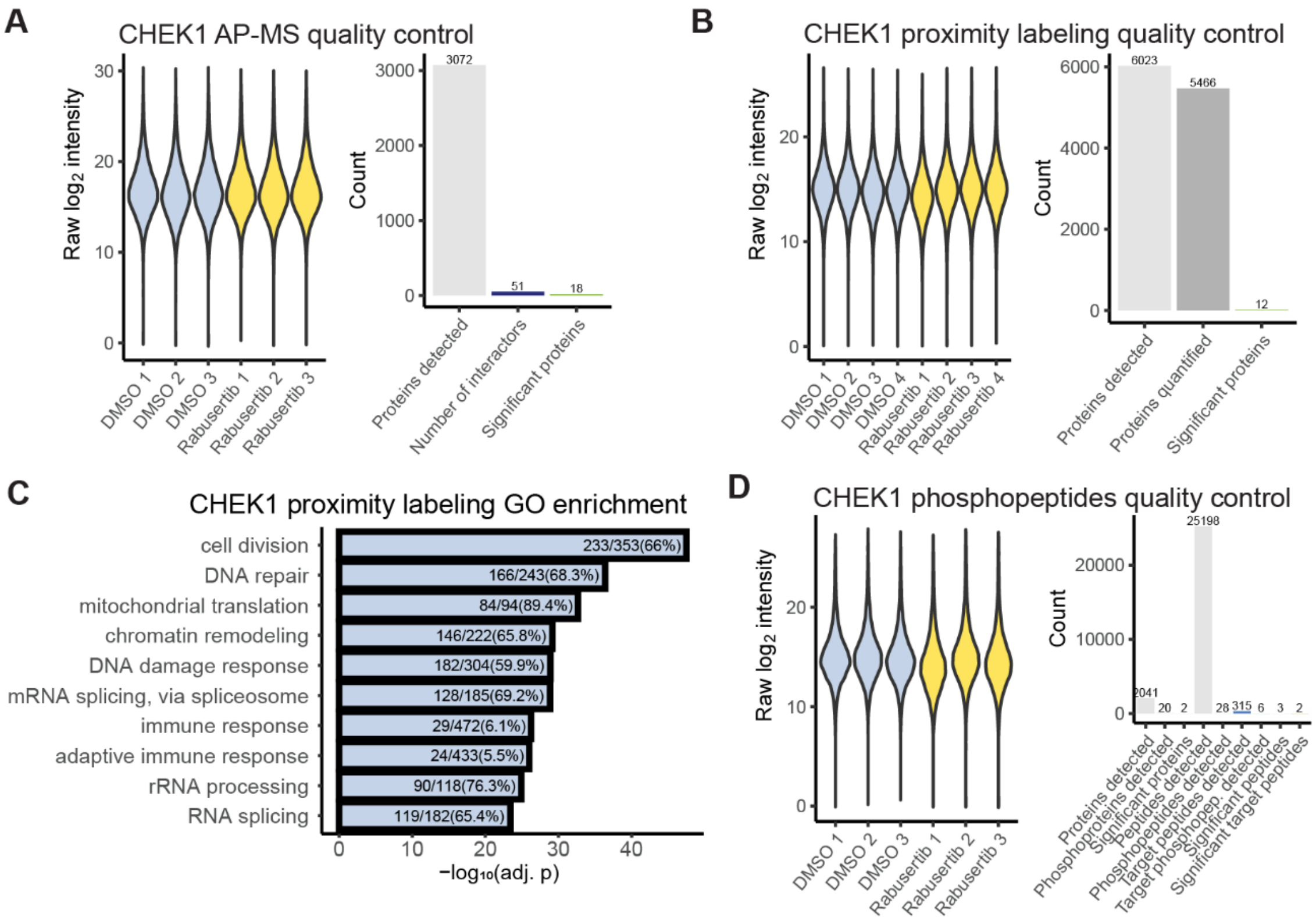


**Appendix Figure S6. Quality control for characterization of CHEK1 complexes and proximity upon rabusertib treatment relating to Fig. 4.** A, B) Raw intensities and summary of metrics related to the proteins quantified for CHEK1 AP-MS and *in vivo* proximity labeling, respectively. C) GO enrichment analysis (biological process) across all proteins quantified in the *in vivo* proximity labeling experiment using CHEK1 as bait. Only enriched terms were plotted. P-values were calculated using a Fisher's exact test followed by multiple testing corrections using the procedure of Benjamini-Hochberg. D) Raw intensities and summary of metrics related to the peptides quantified for CHEK1 phosphopeptide analysis. AP-MS experiments were performed in biological triplicates, and *in vivo* proximity labeling experiments in biological quadruplicates.

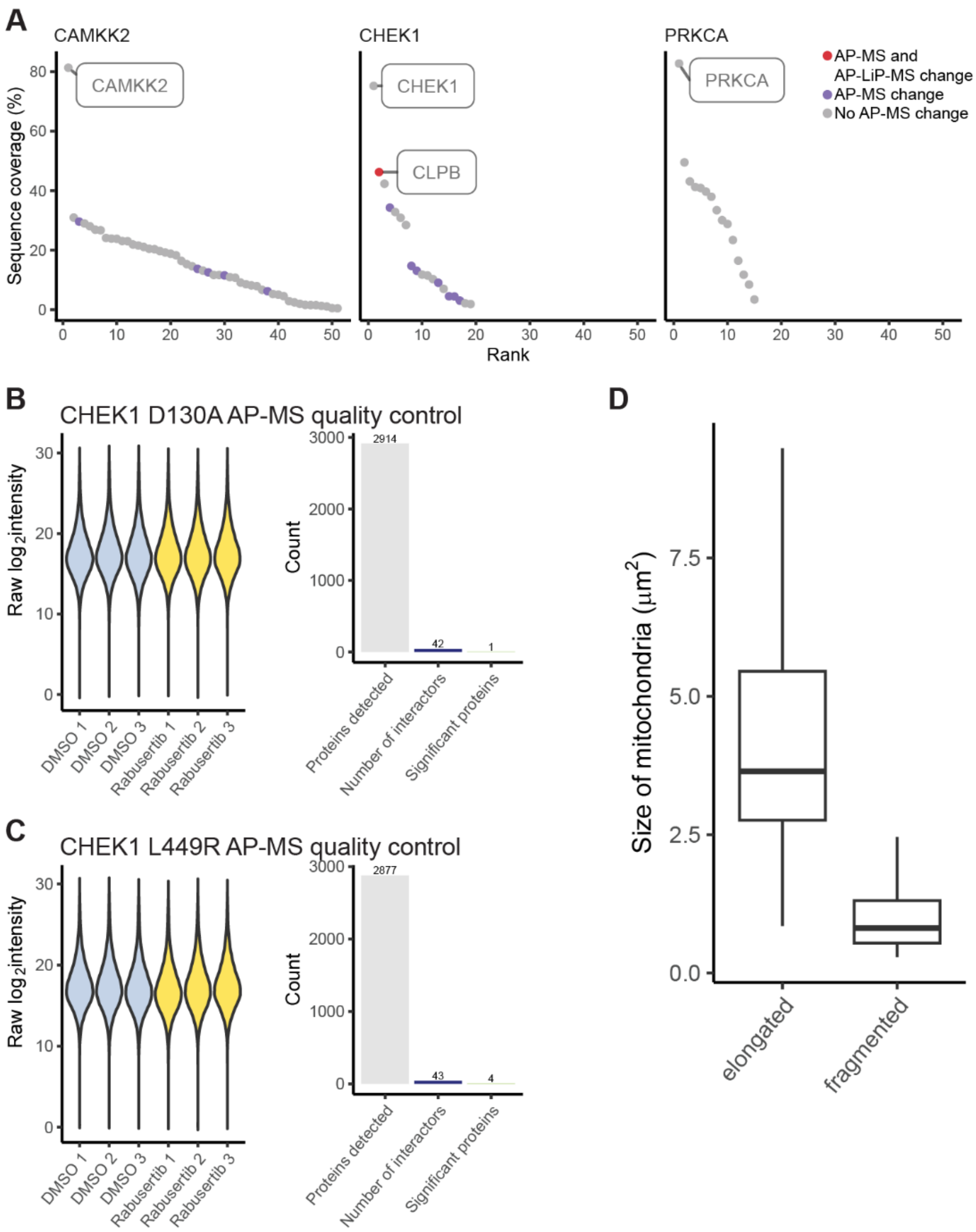


**Appendix Figure S7. Quality control for characterization of CHEK1-CLPB complexes and mitochondrial morphology relating to Fig. 5.** A) Overlap between AP-LiP-MS and AP-MS changes ranked by coverage in AP-LiP-MS experiments. CLPB is the only interactor that shows a structural change by AP-LiP-MS and an interaction change by AP-MS. B, C) Raw intensities and summary of metrics related to the proteins quantified for CHEK1 D130A or L449R AP-MS, respectively. D) Differences in mitochondrial area based on classification as elongated or fragmented. Note that mitochondria were not classified only based on size, but also on shape, branching and other factors. All AP-MS experiments were performed in biological triplicates.

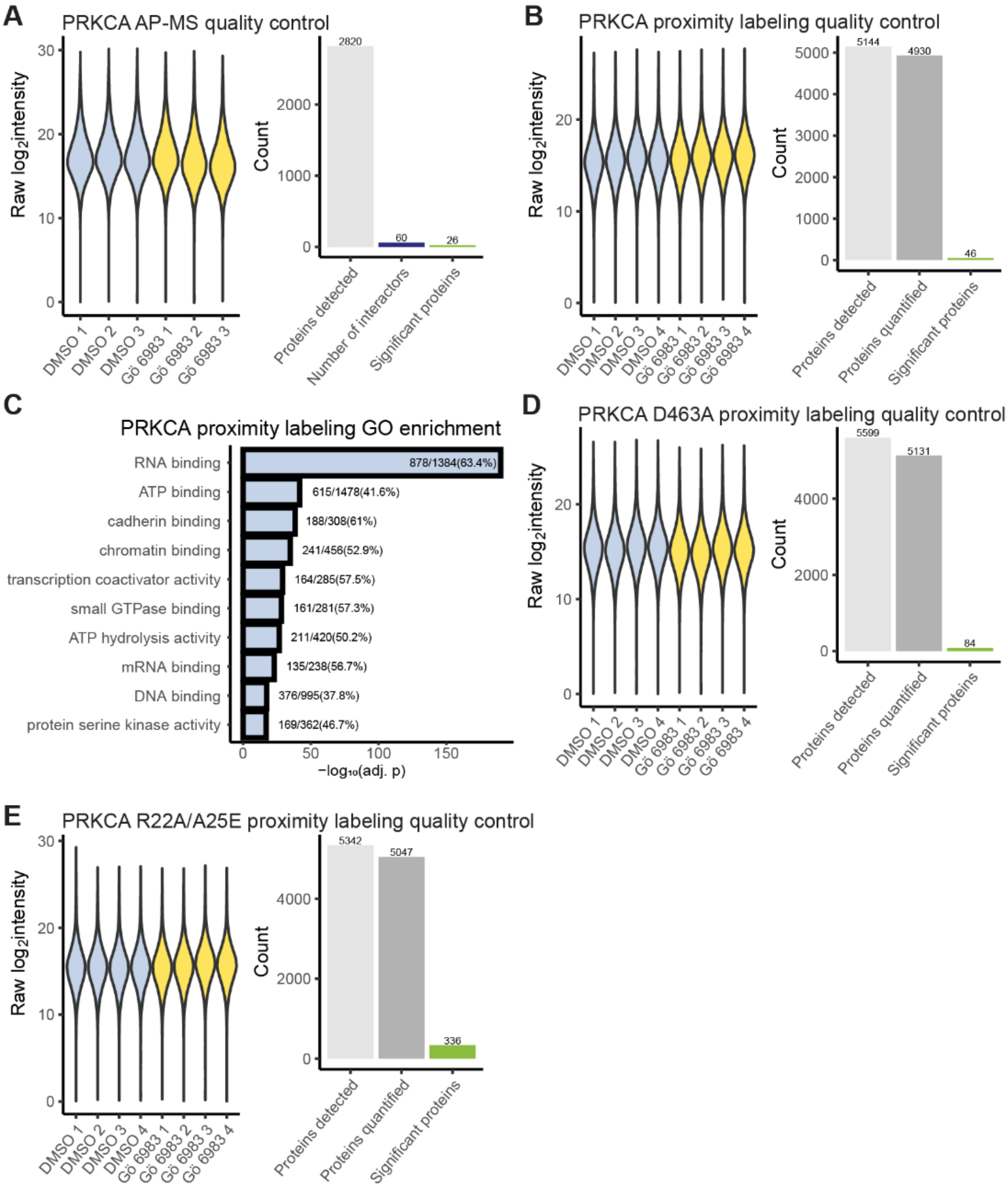


**Appendix Figure S8. Quality control of characterization of the relocalization of PRKCA upon Gö 6983 treatment relating to Fig. 6.** A, B) Raw intensities and summary of metrics related to the proteins quantified for PRKCA AP-MS and *in vivo* proximity labeling, respectively. C) GO enrichment analysis (molecular function) across all proteins quantified in the *in vivo* proximity labeling experiment using PRKCA as bait. Only enriched terms were plotted. P-values were calculated using a Fisher's exact test followed by multiple testing corrections using the procedure of Benjamini-Hochberg. D, E) Raw intensities and summary of metrics related to the proteins quantified for PRKCA D463A and R22A/A25E *in vivo* proximity labeling, respectively. AP-MS experiments were performed in biological triplicates, and *in vivo* proximity labeling experiments in biological quadruplicates.

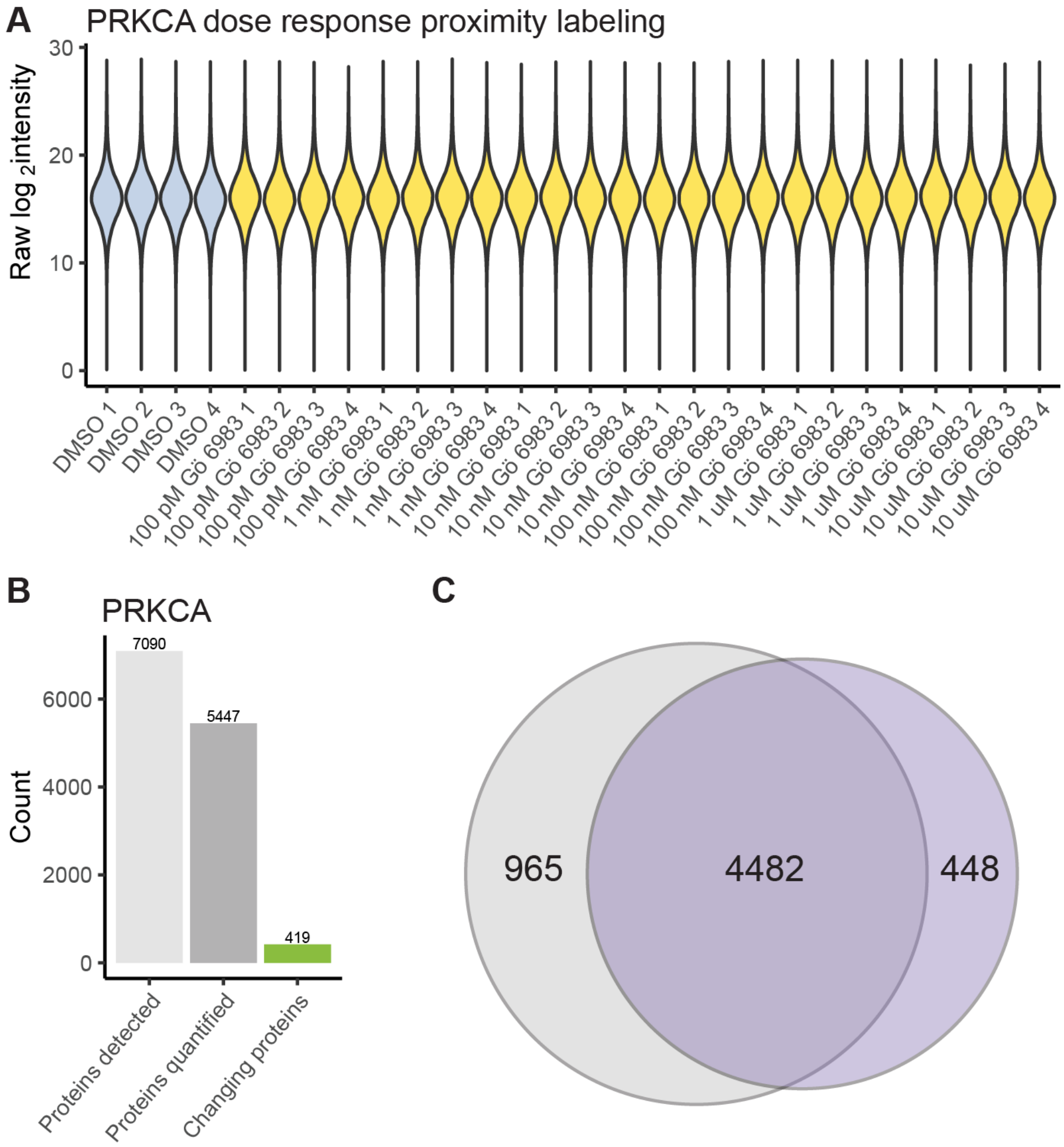


**Appendix Figure S9. Quality control of dose-response proximity labeling for PRKCA upon Gö 6983 treatment relating to Fig. EV4.** A) Raw intensities related to the proteins quantified for PRKCA *in vivo* proximity labeling. B) Summary of metrics related to the proteins quantified for PRKCA *in vivo* proximity labeling. C) Overlap between quantified proteins for dose-response *in vivo* proximity labeling (grey) compared to PRKCA WT single dose *in vivo* proximity labeling (purple). All proximity labeling experiments were performed in biological quadruplicates.

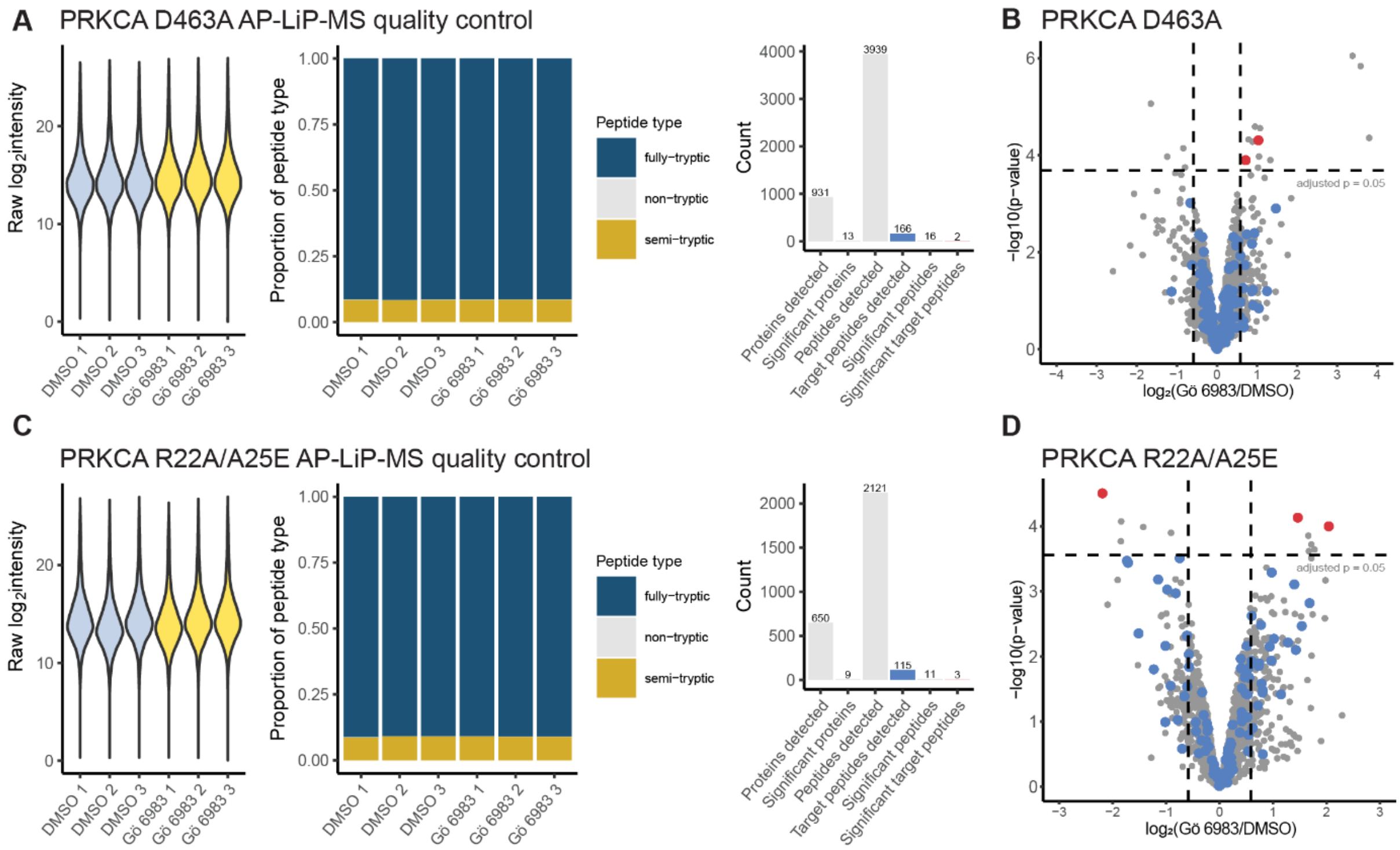


**Appendix Figure S10. Quality control of AP-LiP-MS experiments relating to Fig. EV4.** A, C) Raw intensities, raw peptide type distribution, and summary of metrics related to the peptides quantified for PRKCA D463A and R22A/A25E, respectively. B, D) Volcano plot showing LiP peptide intensity changes in the kinase-enriched samples for PRKCA D463A and R22A/A25E, respectively, upon treatment with Gö 6983 compared to vehicle control. In red are significantly changing target peptides, in blue are the non-changing target peptides. Peptides from other proteins are shown in grey. All AP-LiP-MS experiments were performed in technical triplicates and p-values were calculated using a moderated t-test followed by multiple testing corrections using the procedure of Benjamini-Hochberg.

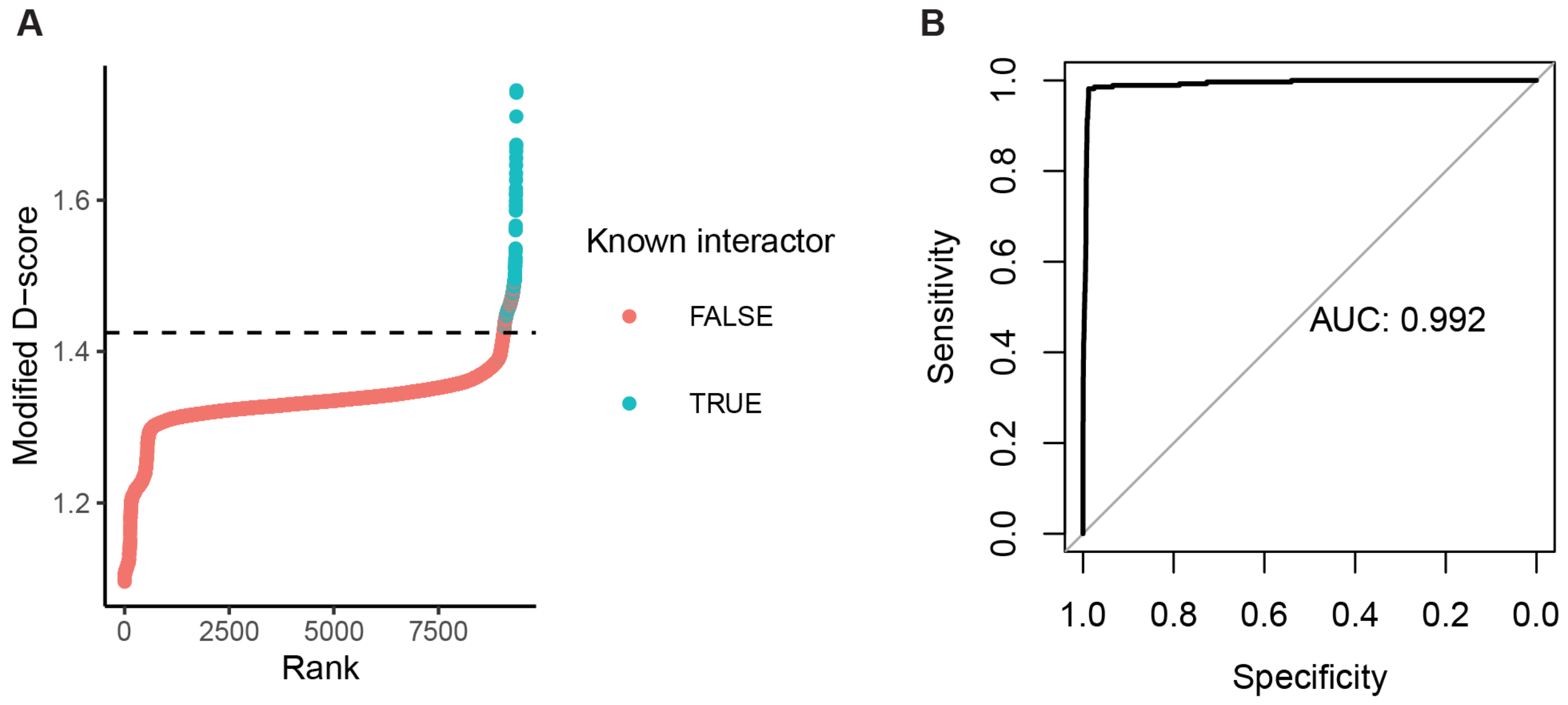


**Appendix Figure S11. Determination of interactors using a modified WD score for AP-MS data.** A) Ranking of the modified D-score of known and unknown interactors across all baits. The cutoff was set at 1.425. Any protein above this cutoff in either treatment condition was considered a potential interactor. B) ROC AUC analysis of the modified D-score predicting known interactors across all baits. The AUC is 0.992. The diagonal reference line shows AUC = 0.5.

**Appendix Table S1: Sequence coverage of kinases across relevant domains relating to Fig. 1.** Shown is the sequence coverage for the entire sequence, the kinase domain (KD) and the autoinhibitory domain (AID).

| Kinase | Total sequence coverage | KD sequence coverage | AID Sequence coverage |
|---|---|---|---|
| DCLK1 | 75.4% | 64.9% | 93.5% |
| CAMKK2 | 81.3% | 77.3% | 86.2% |
| CHEK1 | 75.2% | 79.0% | 60.5% |
| PRKCA | 82.7% | 79.2% | 66.7% |

**Appendix Table S2: Interactors of CAMKK2 identified by AP-MS across all treatment conditions.** Interactors were determined by bait and treatment condition using a modified version of the weighted D-score (see Methods for details). Any protein with a score above 1.425 was considered an interactor.

| Gene name | UniProt Accession | Known BioGrid Interactor | Maximal score |
|---|---|---|---|
| ACAA2 | P42765 | FALSE | 1.44024 |
| AFG3L2 | Q9Y4W6 | FALSE | 1.473367 |
| ALDH3A2 | P51648 | FALSE | 1.46762 |
| ARAF | P10398 | FALSE | 1.456088 |
| ATP1B3 | P54709 | FALSE | 1.437549 |
| CAMKK2 | Q96RR4 | TRUE | 1.745324 |
| CDC37 | Q16543 | TRUE | 1.518405 |
| CDC42EP1 | Q00587 | FALSE | 1.490695 |
| COPB1 | P53618 | FALSE | 1.463998 |
| CTSB | P07858 | FALSE | 1.445539 |
| DCAF8 | Q5TAQ9 | FALSE | 1.46323 |
| DOP1B | Q9Y3R5 | FALSE | 1.448148 |
| DYNC1LI2 | O43237 | FALSE | 1.450658 |
| ECPAS | Q5VYK3 | FALSE | 1.442603 |
| ESYT2 | A0FGR8 | TRUE | 1.562158 |
| FAM83H | Q6ZRV2 | FALSE | 1.476072 |
| FANCD2 | Q9BXW9 | FALSE | 1.427346 |
| FANCI | Q9NVI1 | TRUE | 1.481436 |
| FGF2 | P09038 | FALSE | 1.430508 |
| FLNA | P21333 | TRUE | 1.45766 |
| FOXK2 | Q01167 | FALSE | 1.432111 |
| GCN1 | Q92616 | TRUE | 1.500203 |
| GGCX | P38435 | FALSE | 1.456247 |
| HIP1R | O75146 | FALSE | 1.441217 |
| HK1 | P19367 | FALSE | 1.494657 |
| HSP90AA1 | P07900 | TRUE | 1.494427 |
| HSPA2 | P54652 | FALSE | 1.465704 |
| HSPA4L | O95757 | TRUE | 1.471913 |
| IQGAP2 | Q13576 | TRUE | 1.467299 |
| IRAK1 | P51617 | TRUE | 1.482286 |
| KNTC1 | P50748 | FALSE | 1.445306 |
| MARK4 | Q96L34 | FALSE | 1.456161 |
| MON2 | Q7Z3U7 | FALSE | 1.474501 |
| MYO6 | Q9UM54 | FALSE | 1.445281 |
| NDUFS7 | O75251 | FALSE | 1.479526 |
| NF1 | P21359 | FALSE | 1.426087 |
| NPEPPS | P55786 | FALSE | 1.43914 |
| OBSL1 | O75147 | TRUE | 1.531543 |
| PARK7 | Q99497 | TRUE | 1.457196 |
| PFKM | P08237 | FALSE | 1.473358 |
| PPP6C | O00743 | FALSE | 1.467793 |
| PPP6R3 | Q5H9R7 | FALSE | 1.471 |
| PRKAA1 | Q13131 | TRUE | 1.655716 |
| PRKAA2 | P54646 | TRUE | 1.62695 |
| PRKAG1 | P54619 | TRUE | 1.671049 |
| PRKDC | P78527 | TRUE | 1.471861 |
| SCO2 | O43819 | FALSE | 1.466272 |
| SLC25A1 | P53007 | TRUE | 1.471115 |
| SLC25A11 | Q02978 | TRUE | 1.490104 |
| SLC3A2 | P08195 | FALSE | 1.453671 |
| SMC1A | Q14683 | TRUE | 1.464657 |
| SMC3 | Q9UQE7 | TRUE | 1.459327 |

| | | | |
|---|---|---|---|
| SPTLC1 | O15269 | FALSE | 1.465125 |
| STON2 | Q8WXE9 | FALSE | 1.464973 |
| TANGO6 | Q9C0B7 | FALSE | 1.445534 |
| THAP11 | Q96EK4 | TRUE | 1.483681 |
| TM9SF3 | Q9HD45 | FALSE | 1.466035 |
| TMEM209 | Q96SK2 | FALSE | 1.444921 |
| TMEM263 | Q8WUH6 | FALSE | 1.468814 |
| TUBGCP2 | Q9BSJ2 | FALSE | 1.482299 |
| UBE3C | Q15386 | FALSE | 1.449353 |
| VPS4A | Q9UN37 | FALSE | 1.445089 |
| XPOT | O43592 | TRUE | 1.488375 |
| ZMAT5 | Q9UDW3 | FALSE | 1.475343 |
| ZNF174 | Q15697 | FALSE | 1.478126 |
| ZNF92 | Q03936 | FALSE | 1.493794 |

**Appendix Table S3: Sequence coverage of CAMKK2 mutants across relevant domains relating to Fig. 3A.** Shown is the sequence coverage for the entire sequence, the kinase domain (KD) and the autoinhibitory domain (AID). The sequence coverage for CAMKK2 WT has already been reported in Appendix Table S1.

| Kinase | Total sequence coverage | KD sequence coverage | AID Sequence coverage |
|---|---|---|---|
| CAMKK2 WT | 81.3% | 77.3% | 86.2% |
| CAMKK2 D312A | 78.4% | 73.8% | 65.5% |
| CAMKK2 T483D | 81.0% | 78.7% | 65.5% |
| CAMKK2 R311C | 79.1% | 75.2% | 69.0% |

**Appendix Table S4: Interactors of CHEK1 identified by AP-MS across all treatment conditions.** Interactors were determined by bait and treatment condition using a modified version of the weighted D-score (see Methods for details). Any protein with a score above 1.425 was considered an interactor.

| Gene name | UniProt Accession | Known BioGrid Interactor | Maximal score |
|---|---|---|---|
| BLM | P54132 | TRUE | 1.449943 |
| CAPZB | P47756 | TRUE | 1.454838 |
| CCDC12 | Q8WUD4 | FALSE | 1.480635 |
| CDC37 | Q16543 | TRUE | 1.486203 |
| CDC5L | Q99459 | TRUE | 1.476245 |
| CDK1 | P06493 | TRUE | 1.472145 |
| CHEK1 | O14757 | TRUE | 1.664952 |
| CLPB | Q9H078 | TRUE | 1.615227 |
| CSNK2B | P67870 | TRUE | 1.456171 |
| CTBP1 | Q13363 | FALSE | 1.464498 |
| CUL4A | Q13619 | TRUE | 1.479329 |
| CWC22 | Q9HCG8 | FALSE | 1.431847 |
| DCAF8 | Q5TAQ9 | FALSE | 1.425562 |
| DDB1 | Q16531 | TRUE | 1.491276 |
| FUS | P35637 | TRUE | 1.454455 |
| H1-10 | Q92522 | TRUE | 1.513278 |
| HMGB3 | O15347 | FALSE | 1.446154 |
| HSP90AA1 | P07900 | TRUE | 1.488287 |
| LSM3 | P62310 | FALSE | 1.483499 |
| MCM2 | P49736 | TRUE | 1.449287 |
| MCM3 | P25205 | TRUE | 1.485414 |
| MCM4 | P33991 | TRUE | 1.438203 |
| MCM5 | P33992 | TRUE | 1.448998 |
| MCM6 | Q14566 | TRUE | 1.455789 |
| MEN1 | O00255 | TRUE | 1.472779 |
| MSH6 | P52701 | TRUE | 1.442098 |
| NPM1 | P06748 | TRUE | 1.499068 |
| NPM3 | O75607 | FALSE | 1.440408 |
| PARP1 | P09874 | TRUE | 1.485756 |
| PCNA | P12004 | TRUE | 1.464558 |
| PM20D2 | Q8IYS1 | FALSE | 1.440793 |
| PPM1G | O15355 | FALSE | 1.428902 |
| PRKDC | P78527 | TRUE | 1.461032 |
| PSMD4 | P55036 | FALSE | 1.451336 |
| RACGAP1 | Q9H0H5 | TRUE | 1.456254 |
| RPS27A | P62979 | TRUE | 1.521202 |
| SNW1 | Q13573 | TRUE | 1.458479 |
| SRM | P19623 | FALSE | 1.472898 |
| TOM1 | O60784 | TRUE | 1.467789 |
| TP53 | P04637 | TRUE | 1.457303 |
| UBA1 | P22314 | TRUE | 1.434492 |
| USP7 | Q93009 | TRUE | 1.460129 |
| VCP | P55072 | TRUE | 1.453499 |
| WDR48 | Q8TAF3 | FALSE | 1.439921 |
| XRCC5 | P13010 | TRUE | 1.469176 |
| XRCC6 | P12956 | TRUE | 1.463784 |
| YWHAB | P31946 | TRUE | 1.47423 |
| YWHAG | P61981 | TRUE | 1.460321 |

| | | | |
|---|---|---|---|
| YWHAH | Q04917 | TRUE | 1.459052 |
| YWHAQ | P27348 | TRUE | 1.467858 |
| YWHAZ | P63104 | TRUE | 1.460007 |

**Appendix Table S5: Sequence coverage of PRKCA mutants across relevant domains relating to Fig. EV4A.** Shown is the sequence coverage for the entire sequence, the kinase domain (KD), the autoinhibitory domain (AID) and the C2 domain. The sequence coverages for the KD and AID for PRKCA WT have already been reported in Appendix Table S1.

| Kinase | Total sequence coverage | KD sequence coverage | AID Sequence coverage | C2 domain sequence coverage |
|---|---|---|---|---|
| PRKCA | 82.7% | 79.2% | 66.7% | 88.1% |
| PRKCA D463A | 67.0% | 67.2% | 23.8% | 78.0% |
| PRKCA R22A/A25E | 62.5% | 54.1% | 23.8% | 83.9% |

**Appendix Table S6: List of used doxycycline-inducible expression vectors and their source.**

| Name | Source |
|---|---|
| N-pTOSH-DCLK1 | Buljan M. *et al*, *Mol. Cell*, 2020 |
| N-miniTurbo-CAMKK2 | pDONR223-CAMKK2 (addgene 23422) |
| N-pTOSH-CAMKK2 | pDONR223-CAMKK2 (addgene 23422) |
| N-pTOSH-CAMKK2 R311C | Mutated from pDONR223-CAMKK2 |
| N-pTOSH-CAMKK2 D312A | Mutated from pDONR223-CAMKK2 |
| N-pTOSH-CAMKK2 T483D | Mutated from pDONR223-CAMKK2 |
| N-pTOSH-CHEK1 | Buljan M. *et al*, *Mol. Cell*, 2020 |
| N-pTOSH-CHEK1 D130A | Mutated from N-pTOSH-CHEK1 |
| N-pTOSH-CHEK1 L449R | Mutated from N-pTOSH-CHEK1 |
| N-miniTurbo-CHEK1 | pDONR221-CHEK1 (Varjosalo M. *et al*, *Cell*, 2008) |
| N-pTOSH-PRKCA wt | pDONR223-PRKCA (ORFeome 51011@B05) |
| N-pTOSH-PRKCA R22A/A25E | Mutated from pDONR223-PRKCA |
| N-pTOSH-PRKCA D463A | Mutated from pDONR223-PRKCA |
| N-EGFP- PRKCA wt | pDONR223-PRKCA (ORFeome 51011@B05) |
| N-miniTurbo-PRKCA wt | pDONR223-PRKCA (ORFeome 51011@B05) |
| N-miniTurbo-PRKCA R22A/A25E | Mutated from pDONR-PRKCA |
| N-miniTurbo-PRKCA D463A | Mutated from pDONR-PRKCA |
| N-miniTurbo-PRKAA1 | pDONR223-PRKAA1 (ORFeome 31002@A11) |
| N-miniTurbo-EGFP | pDONR221_EGFP (addgene 25899) |
| N-pTOSH-EGFP | pDONR221_EGFP (addgene 25899) |

**Appendix Table S7: List of primers used for site directed mutagenesis.**

| Gene | Name | Sequence: (5' to 3') |
|---|---|---|
| CAMKK2 | R311C forward | CTACCAGAAGATCATCCACTGCGACATCAAACCTTCCAACCTCCTGGTCGGAG |
| CAMKK2 | R311C reverse | CTCCGACCAGGAGGTTGGAAGGTTTGATGTCGCAGTGGATGATCTTCTGGTAG |
| CAMKK2 | D312A forward | CCAGAAGATCATCCACCGTGCGATCAAACCTTCCAACCTCCTGGTCGGAGAAG |
| CAMKK2 | D312A reverse | CTTCTCCGACCAGGAGGTTGGAAGGTTTGATCGCACGGTGGATGATCTTCTGG |
| CAMKK2 | T483D forward | CAAACACATTCCCAGCTTGGCAGATGTGATCCTGGTGAAGACC |
| CAMKK2 | T483D reverse | GGTCTTCACCAGGATCACATCTGCCAAGCTGGGAATGTGTTTG |
| CHEK1 | D131A forward | GCATGGTATTGGAATAACTCACAGGGCGATTAAACCAG |
| CHEK1 | D131A reverse | CTGGTTTAATCGCCCTGTGAGTTATTCCAATACCATGC |
| CHEK1 | L449R forward | CATGACGTTATCTAACTTCAGGGCCCTATAAATGATTCCTC |
| CHEK1 | L449R reverse | GGAAGTGTCTCTTGAACTCACGTCCATCACCCTTAGAAAGCCG |
| PRKCA | R22A/A25E forward | CCGCTTCGCCGCCAAAGGGGAGCTGAGGCAG |
| PRKCA | R22A/A25E reverse | CTGCCTCAGCTCCCCTTTGGCGGCGAAGCGG |
| PRKCA | D463A forward | GAGGAATCATTTATAGGGCCCTGAAGTTAGATAACGTCATG |
| PRKCA | D463A reverse | CATGACGTTATCTAACTTCAGGGCCCTATAAATGATTCCTC |

**Appendix Table S8: Isolation windows used for DIA methods on the Orbitrap Fusion Lumos Tribrid mass spectrometer.**

| Window | Center m/z | z | Isolation Window size (m/z) |
|---|---|---|---|
| 1 | 358 | 2 | 16 |
| 2 | 373 | 2 | 16 |
| 3 | 388 | 2 | 16 |
| 4 | 403 | 2 | 16 |
| 5 | 418 | 2 | 16 |
| 6 | 433 | 2 | 16 |
| 7 | 448 | 2 | 16 |
| 8 | 463 | 2 | 16 |
| 9 | 478 | 2 | 16 |
| 10 | 493 | 2 | 16 |
| 11 | 508 | 2 | 16 |
| 12 | 523 | 2 | 16 |
| 13 | 538 | 2 | 16 |
| 14 | 553 | 2 | 16 |
| 15 | 568 | 2 | 16 |
| 16 | 583 | 2 | 16 |
| 17 | 598 | 2 | 16 |
| 18 | 613 | 2 | 16 |
| 19 | 628 | 2 | 16 |
| 20 | 643 | 2 | 16 |
| 21 | 659 | 2 | 18 |
| 22 | 676 | 2 | 18 |
| 23 | 693 | 2 | 18 |
| 24 | 710 | 2 | 18 |
| 25 | 727 | 2 | 18 |
| 26 | 744 | 2 | 18 |
| 27 | 761 | 2 | 18 |
| 28 | 778 | 2 | 18 |
| 29 | 795 | 2 | 18 |
| 30 | 813 | 2 | 20 |
| 31 | 832 | 2 | 20 |
| 32 | 851 | 2 | 20 |
| 33 | 870 | 2 | 20 |
| 34 | 889 | 2 | 20 |
| 35 | 908 | 2 | 20 |
| 36 | 929.5 | 2 | 25 |
| 37 | 953.5 | 2 | 25 |
| 38 | 977.5 | 2 | 25 |
| 39 | 1006.5 | 2 | 35 |
| 40 | 1048 | 2 | 50 |
| 41 | 1111 | 2 | 78 |

**Appendix Table S9: Isolation windows used for DIA methods on the Orbitrap Exploris 480 mass spectrometer.**

| Number | Lower bound [m/z] | Upper bound [m/z] | Window size [m/z] |
|---|---|---|---|
| 1 | 350 | 366 | 16 |
| 2 | 365 | 381 | 16 |
| 3 | 380 | 397 | 17 |
| 4 | 396 | 411 | 15 |
| 5 | 410 | 426 | 16 |
| 6 | 425 | 441 | 16 |
| 7 | 440 | 456 | 16 |
| 8 | 455 | 471 | 16 |
| 9 | 470 | 486 | 16 |
| 10 | 485 | 501 | 16 |
| 11 | 500 | 516 | 16 |
| 12 | 515 | 531 | 16 |
| 13 | 530 | 546 | 16 |
| 14 | 545 | 561 | 16 |
| 15 | 560 | 576 | 16 |
| 16 | 575 | 591 | 16 |
| 17 | 590 | 606 | 16 |
| 18 | 605 | 621 | 16 |
| 19 | 620 | 636 | 16 |
| 20 | 635 | 651 | 16 |
| 21 | 650 | 668 | 18 |
| 22 | 667 | 685 | 18 |
| 23 | 684 | 702 | 18 |
| 24 | 701 | 719 | 18 |
| 25 | 718 | 736 | 18 |
| 26 | 735 | 753 | 18 |
| 27 | 752 | 770 | 18 |
| 28 | 769 | 787 | 18 |
| 29 | 786 | 804 | 18 |
| 30 | 803 | 823 | 20 |
| 31 | 822 | 842 | 20 |
| 32 | 841 | 861 | 20 |
| 33 | 860 | 880 | 20 |
| 34 | 879 | 899 | 20 |
| 35 | 898 | 918 | 20 |
| 36 | 917 | 942 | 25 |
| 37 | 941 | 966 | 25 |
| 38 | 965 | 990 | 25 |
| 39 | 989 | 1024 | 35 |
| 40 | 1023 | 1073 | 50 |
| 41 | 1072 | 1150 | 78 |

**Appendix Table S10: Sequences used for the prediction of the CAMKK2-PRKAA1 complex.** Sequences correspond to the kinase domains as annotated on UniProt[1].

| Gene | Accession | Sequence |
|---|---|---|
| CAMKK2 | Q96RR4 | YTLKDEIGKGSYGVVKLAYNENDNTYYAMKVLSKKKLIRQAGFPRRPPPRGTRP<br>APGGCIQPRGPIEQVYQEIAILKKLDHPNVVKLVEVLDDPNEDHLYMVFELVNQ<br>GPVMEVPTLKPLSEDQARFYFQDLIKGIEYLHYQKIIHRDIKPSNLLVGEDGHIKIA<br>DFGVSNEFKGSDALLSNTVGTPAFMAPESLSETRKIFSGKALDVWAMGVTLYC<br>FVFGQCPFMDERIMCLHSKIKSQALEFPDQPDIAEDLKDLITRMLDKNPESRIVV<br>PEIKLHPWV |
| PRKAA1 | Q13131 | YILGDTLGVGTFGKVKVGKHELTGHKVAVKILNRQKIRSLDVVGKIRREIQNLKLF<br>RHPHIIKLYQVISTPSDIFMVMEYVSGGELFDYICKNGRLDEKESRRLFQQILSGV<br>DYCHRHMVVHRDLKPENVLLDAHMNAKIADFGLSNMMSDGEFLRTSCGSPNY<br>AAPEVISGRLYAGPEVDIWSSGVILYALLCGTLPFDDDHVPTLFKKICDGIFYTPQ<br>YLNPSVISLLKHMLQVDPMKRATIKDIREHEWF |


1. UniProt, C. UniProt: the Universal Protein Knowledgebase in 2025. *Nucleic Acids Res* **53**, D609–D617 (2025).
