# Supplementary material for: Paradoxical non-catalytic kinase functions are driven by inhibitor-induced displacement of autoinhibitory domains
Source: Mol Syst Biol. 2026 Jul 2;22(9):1474–500. doi: 10.1038/s44320-026-00229-2 (PMC13538393; doi:10.1038/s44320-026-00229-2)
Supplement: Supplementary file 3 [file 44320_2026_229_MOESM2_ESM.pdf]

molecular systems biology

Paradoxical non-catalytic kinase functions are driven by inhibitor-induced displacement of autoinhibitory domains

Viviane Reber, Sabrina Keller, Stefanie Loosli, Yumi Arima, Tatjana Kleele, Paola Picotti, and Matthias Gstaiger

Corresponding author(s): Matthias Gstaiger (matthias.gstaiger@imsb.biol.ethz.ch)

Review Timeline:

Submission Date:	2nd Mar 26
Editorial Decision:	3rd Apr 26
Revision Received:	5th Jun 26
Editorial Decision:	15th Jun 26
Revision Received:	16th Jun 26
Accepted:	18th Jun 26

Editor: Jingyi Hou

Transaction Report:

(Note: With the exception of the correction of typographical or spelling errors that could be a source of ambiguity, letters and reports are not edited. Depending on transfer agreements, referee reports obtained elsewhere may or may not be included in this compilation. Referee reports are anonymous unless the Referee chooses to sign their reports.)

3rd Apr 2026

Manuscript Number: MSB-2026-13670
Title: Paradoxical non-catalytic kinase functions are driven by inhibitor-induced displacement of autoinhibitory domains
Author: Viviane Reber
Sabrina Keller
Stefanie Loosli
Yumi Arima
Tatjana Kleele
Paola Picotti
Matthias Gstaiger

Dear Dr. Gstaiger,

Thank you again for submitting your work to Molecular Systems Biology. We have now heard back from the three reviewers who agreed to evaluate your manuscript. As you will see from the reports below, the reviewers find the study interesting and relevant. They raise, however, a series of concerns, which should be convincingly addressed in a major revision.

The reviewers' recommendations are relatively clear, so there is no need to reiterate the points listed below. All the issues raised by the reviewers need to be satisfactorily addressed. As you may already know, our editorial policy allows in principle a single round of major revision, and it is therefore essential to provide responses to the reviewers' comments that are as complete as possible. Please feel free to contact me in case you would like to discuss in further detail any of the issues raised by the reviewers.

On a more editorial level, we would ask you to address the following issues:

- Please provide a .docx formatted version of the manuscript text (including legends for main figures, EV figures and tables). Please make sure that the changes are highlighted to be clearly visible.

- Please provide individual production quality figure files as .eps, .tif, .jpg (one file per figure). When assembling figures, please refer to our figure preparation guideline in order to ensure proper formatting and readability in print as well as on screen: https://media.springernature.com/original/springer-cms/rest/v1/content/27825798/data/v1

-Please provide a .docx formatted letter INCLUDING the reviewers' reports and your detailed point-by-point responses to their comments. As part of the EMBO Press transparent editorial process, the point-by-point response is part of the Review Process File (RPF), which will be published alongside your paper.

-Please note that all corresponding authors are required to supply an ORCID ID for their name upon submission of a revised manuscript.

-We replaced Supplementary Information with Expanded View (EV) Figures and Tables that are collapsible/expandable online. A maximum of 5 EV Figures can be typeset. EV Figures should be cited as 'Figure EV1, Figure EV2" etc... in the text and their respective legends should be included in the main text after the legends of regular figures.

Additional Tables/Datasets should be labeled and referred to as Table EV1, Dataset EV1, etc. Legends have to be provided in a separate tab in case of .xls files. Alternatively, the legend can be supplied as a separate text file (README) and zipped together with the Table/Dataset file.

For the figures and tables that you do NOT wish to display as Expanded View figures, they should be bundled together with their legends in a single PDF file called *Appendix*, which should start with a short Table of Content. Each legend should be below the corresponding Figure/Table in the Appendix. Appendix figures and tables should be referred to in the main text as: "Appendix Figure S1, Appendix Figure S2, Appendix Table S1" etc. See detailed instructions regarding expanded view here: https://link.springer.com/journal/44320/submission-guidelines#cms-Expanded-View-data.

-Before submitting your revision, primary datasets (and computer code, where appropriate) produced in this study need to be deposited in an appropriate public database.
Please remember to provide a reviewer password if the datasets are not yet public.
The accession numbers and database should be listed in a formal "Data Availability" section (placed after Materials & Method) that follows the model below (see also https://link.springer.com/partners/embo-press/editorial-policies#Data%20availability%20statement). Please note that the Data Availability Section is restricted to new primary data that are part of this study.

Data availability
The datasets (and computer code) produced in this study are available in the following databases:
- RNA-Seq data: Gene Expression Omnibus GSE46843 (https://www.ncbi.nlm.nih.gov/geo/query/acc.cgi?acc=GSE46843)
- [data type]: [name of the resource] [accession number/identifier/doi] ([URL or identifiers.org/DATABASE:ACCESSION])

*** Note - All links should resolve to a page where the data can be accessed. ***

- At EMBO Press we ask authors to provide source data for the main manuscript figures. You will receive a separate email with instructions for providing source data with your revised manuscript, including how to upload and organize the files.
Additional information on source data and instruction on how to label the files are available < https://link.springer.com/journal/44320/submission-guidelines#cms-Source-data >

- Our journal encourages inclusion of *data citations in the reference list* to directly cite datasets that were re-used and obtained from public databases. Data citations in the article text are distinct from normal bibliographical citations and should directly link to the database records from which the data can be accessed. In the main text, data citations are formatted as follows: "Data ref: Smith et al, 2001". In the Reference list, data citations must be labeled with "[DATASET]". A data reference must provide the database name, accession number/identifiers and a resolvable link to the landing page from which the data can be accessed at the end of the reference. Further instructions are available at < https://link.springer.com/journal/44320/submission-guidelines#cms-Reference-guidelines >.

- We updated our journal's competing interests policy in January 2022 and request authors to consider both actual and perceived competing interests. Please review the policy (https://link.springer.com/partners/embo-press/editorial-policies#Competing%20interest%20disclosures) and update your competing interests if necessary.
Please use the heading "Disclosure statement and competing interests".

- All Materials and Methods need to be described in the main text using our 'Structured Methods' format. According to this format, the Methods section includes a Reagents and Tools Table (listing key reagents, experimental models, software and relevant equipment and including their sources and relevant identifiers) followed by a Methods and Protocols section describing the methods, ideally using a step-by-step protocol format. The aim is to facilitate adoption of the methodologies across labs.

Please download and fill our Reagents and Tools Table template (.docx), which you can find in our author guidelines: https://link.springer.com/journal/44320/submission-guidelines#structuredmethods.

When submitting your revised manuscript, please do not include the Reagents and Tools Table in the Methods section of the manuscript but upload it as a separate file choosing the file type "Reagent Table".

-Regarding data quantification:
Please ensure to specify the name of the statistical test used to generate error bars and P values, the number (n) of independent experiments (please specify technical or biological replicates) underlying each data point and the test used to calculate p-values in each figure legend. Discussion of statistical methodology can be reported in the materials and methods section, but figure legends should contain a basic description of n, P and the test applied.
Graphs must include a description of the bars and the error bars (s.d., s.e.m.).
Please also include scale bars in all microscopy images.

- Please provide a "standfirst text" summarizing the study in one or two sentences (approximately 250 characters, including space), three to four "bullet points" highlighting the main findings and a " visual abstract" (exactly 550px width and 300 px height, PNG format) to highlight the paper on our homepage.

When you resubmit your manuscript, please download our CHECKLIST (https://media.springernature.com/original/springer-cms/rest/v1/content/27825796/data/v1) and include the completed form in your submission.
Please note that the Author Checklist will be published alongside the paper as part of the transparent process .

If you feel you can satisfactorily deal with these points and those listed by the referees, you may wish to submit a revised version of your manuscript. Please attach a covering letter giving details of the way in which you have handled each of the points raised by the referees. A revised manuscript will be once again subject to review and you probably understand that we can give you no guarantee at this stage that the eventual outcome will be favorable.

I look forward to receiving the revised manuscript soon.

Kind regards,
Jingyi

Jingyi Hou, PhD
Senior Editor
Molecular Systems Biology

We realize that it is difficult to revise to a specific deadline. In the interest of protecting the conceptual advance provided by the work, we recommend a revision within 3 months (2nd Jul 2026). Please discuss the revision progress ahead of this time with the editor if you require more time to complete the revisions. Use the link below to submit your revision:

https://msb.msubmit.net/cgi-bin/main.plex

IMPORTANT: Please read our guidelines for revised submissions: https://link.springer.com/journal/44320/submission-guidelines#cms-Revised-submissions

*** PLEASE NOTE *** As part of the EMBO Press transparent editorial process initiative (see our Editorial at https://dx.doi.org/10.1038/msb.2010.72), Molecular Systems Biology publishes online a Review Process File with each accepted manuscripts. This file will be published in conjunction with your paper and will include the anonymous referee reports, your point-by-point response and all pertinent correspondence relating to the manuscript. If you do NOT want this File to be published, please inform the editorial office at contact@molsystbiol.org within 14 days upon receipt of the present letter.

Reviewer #1:

The manuscript "Paradoxical non-catalytic kinase functions are driven by 1 inhibitor-induced displacement of autoinhibitory domains" by Reber et al. described an integrated mass spectrometry approach combining LiP-MS, AP-MS, and proximity labeling-MS to map the inhibitor-induced kinase conformation and interaction changes. This works is very interesting and uncover the widespread mechanisms of kinase inhibitor-induced non-catalytic conformation alterations, which might greatly contribute to the rational design of more effective kinase inhibitors. I think this work can be published on MSB after minor revision.

1. All the Lip-MS experiments were performed after affinity purification. Although this AP approach is more native than in vitro analysis, the sequence and site coverage is a problem. So, can the authors cover all the interesting regions of kinase in ATP binding and autoinhibitory domains? The sequence region coverages for all the protein kinase analysis should be given in the manuscript.
2. How the authors evaluate the kinase conformation changes is directly related to the inhibitor binding or by other protein interactions? As the AP-MS experiments identified many interaction proteins. Maybe some invitro analysis by using purified proteins is still needed?
3. How the in vivo proximity labeling experiment and data analysis are conducted are not clear. They authors should describe the procedures in more detail. If CH_2O is used for in vivo labeling, how to avoid crosslinking, how to do database searching and realize confident results?
4. For AP-Lip-MS analysis, the conformation information of other proteins within the complexes could be also obtained. Can the authors also analysis and discuss these interface alterations.

Reviewer #2:

Summary

Reber et al. address an underexplored dimension of kinase pharmacology: the non-catalytic, "off-mechanism" effects of ATP-competitive inhibitors that is mediated through changes of kinase domain conformation and protein-protein interaction (PPI) changes. The authors develop a powerful proteomics framework using LiP-MS (limited proteolysis mass spectrometry; previously developed by the authors) applied to native afffinity-purified protein complexes, and proximity labeling-MS to systematically map inhibitor-induced structural and interactome remodeling in a native cellular context. Using three Ser/Thr-kinases (CAMKK2, CHEK1, and PRKCA) with distinct autoinhibitory domain (AID) architectures, Reber and colleagues demonstrate that ATP-competitive kinase inhibitor binding induces displacement of the AID, promoting an open, active-like conformation despite catalytic inhibition. This structural transition rewires PPIs and drives diverse (paradoxic) non-catalytic phenotypes. This is nicely demonstrated in relevant cell biological assays for all three kinase-inhibitor pairs. The data is supported by mutational analyses, which allows to disentangle catalytic inhibition from conformational effects. The findings support a model in which the ATP-binding site acts as a central allosteric hub governing kinase conformation and interaction networks.

Overall, the study provides compelling evidence that kinase inhibitor-induced neomorphic PPIs may be a widespread and understudied feature with potentially important biological functions. The proposed proteomics pipeline represents a scalable and generalizable strategy to uncover such effects, with important implications for drug development, particularly in anticipating side effects and improving inhibitor selectivity.

Major comments:
1. While I agree that kinase inhibitor-induced changes of (intra- and intermolecular) PPIs are understudied and some paradoxical effects of kinase inhibitor on signaling are mentioned in the Discussion section (paradoxical MAPK activation by BRAF inhibitors and JAK2 hyperphosphorylation by type I JAK1/2 inhibitors), one very prominent early example is missing and should be included: The Grzesiek lab demonstrated in 2013 that ATP competitive kinase inhibitors targeting BCR::ABL1 result in an open conformation, which enable its SH2 and SH3 domains to engage in PPIs (Skora et al. PNAS 2013; PMID: 24191057).
2. I was a bit puzzled that the authors focus entirely on type I kinase inhibitors. While type I inhibitors are indeed more common and induce an active open conformation of the kinase domain and are therefore prime candidates for the studied phenomenon, it has been shown that also type II inhibitors lead to AID displacement and disassembly of intramolecular PPIs. This was first demonstrated for type II BCR::ABL1 kinase inhibitors (see: Sonti et al. JACS 2018; PMID: 29319304); whereas in contrast type I TKIs (e.g. dasatinib, bosutinib, ...) kept BCR::ABL1 in an assembled conformation. I suggest to include these examples and thereby broaden the scope/impact of the manuscript. Hence, it can be expected that also binding of many type II inhibitors (and potentially also allosteric inhibitors) may also result in conformations that displace AID and induce neomorphic PPIs.
3. The manuscript focuses on kinase inhibitor-induced changes on native (unmutated) kinases. While this is important to study for possible side effects in "off-tissues" or uncover diminished therapeutic effect in the on-target cells/tissues, I lack a discussion and mention that the therapeutic targets of most kinase inhibitors are not native kinases, but active oncogenic kinases that are activated by point mutations, insertions/deletions, duplication, fusion partners etc. These oncogenic kinase variants have often already displaced AIDs, open/disassembled conformations and possibly a different subcellular localization. What is the authors expectation of the consequences of inhibitor-induced (further) changes in this common scenario?

Reviewer #3:

The authors set out to investigate small molecule inhibitor functions beyond catalytic inhibition using a multi-proteomic approach. Focussing on three examples with different structural architecture and three matching selective small molecule inhibitors, structural re-arrangements via LIP-MS and the change of the proximal proteome are investigated upon ligand treatment. The authors identify three separate consequences of ligand binding beyond inhibition: for CHEK1 dissociation from CLBP is identified. This causes mitochondrial fragmentation. PRKCA is re-localised to cell junctions and endosome-like organelles. CAMKK2 triggers AMPK sequestration. The study is overall of very high quality, elucidates novel mechanisms of action beyond inhibition of inhibitors and thus provides timely insights into additional/off-target concequences of small molecule binding. This provides important insights into small molecule actions and further substantiates the need of detailed dissection of inhibitor consequences beyond inhibition. At this point a few open points still, however, remain unclear.

Major points:
• The authors refer multiple times to neomorphic features. This would imply that a novel function of the protein is induced by small molecule binding. However, the authors do not specifically show any neomorphic features and instead it seems like endogenous interactions or functions are modulated by inhibitor binding?
• While the work is of high quality, all three inhibitors have IC50s in the nanomolar range, but are treated at 1 µM. This hampers the strength of the drawn conclusions. Are the observed effects of direct or indirect nature? Could the authors please provide evidence that ligand binding is directly causing the observed non-catalytic effects.
• For PRKCA it would be interesting to see if mutated versions (inactive/constitutively active) occupy the same structural space to WT and how this correlates to the observed behavior upon ligand binding- if the hypothesis is correct Go6983 should lead to the identical structural changes.
• What percentage of loss of PRKCA in native lysis conditions upon Go6983 treatment can be explained by relocalisation to the cellular junctions vs the suggested insoluble organelles?

Minor points:
• Line 17: The abstract lacks a definition of AIDs.
• Line 24: The authors should consider rephrasing "highly prevalent" since here three examples are shown.
• Line 62: naming the inhibitors at this point may be beneficial, including reported EC50s or IC50s.
• Line 111: should include details as to binding affinity/inhibitory capacity and selectivity (see also comment on line 62).
• Line 361: it is unclear in what context "more pervasive feature" is meant.

Molecular Systems Biology, MSB-2026-13670

Paradoxical non-catalytic kinase functions are driven by inhibitor-induced displacement of autoinhibitory domains

Viviane Reber (1), Sabrina Keller (1), Stefanie A. Loosli (1), Yumi Arima (2), Tatjana Kleele (2), Paola Picotti (1), Matthias Gstaiger (1)

1 Institute of Molecular Systems Biology, Department of Biology, ETH Zurich, Zurich, Switzerland

2 Institute of Biochemistry, Department of Biology, ETH Zurich, Zurich, Switzerland

Corresponding author: Matthias Gstaiger, matthias.gstaiger@imsb.biol.ethz.ch

Response to all reviewers:

We thank the reviewers for their supportive comments highlighting the impact and novelty of our study. We greatly appreciate that the reviewers took the time to review our work to provide insightful and constructive feedback that has truly strengthened the manuscript as we understand the time and effort it takes. To address the comments and questions, we have provided new experiments and analyses. In particular, we

- performed structural and interaction proteomics experiments on CAMKK2 using a negative control compound which no longer can bind CAMKK2 to confirm that measured effects depend on direct probe target interaction
- reanalyzed AP-LiP MS data of kinase interactors which revealed rabusertib induced structural changes in the CHEK1 interactor CLPB which helps to explain inhibitor induced mitochondrial fragmentation.
- included new proximity ligation dose response experiments with PRKCA inhibitor Gö 6983 which showed that the claimed changes in PRKCA proximity have a low median EC50 consistent with a direct inhibitor response
- performed a set of new AP-LiP-MS experiments on PRKCA mutants which confirmed structural changes within the PRKCA C2 domain as a main driver of membrane relocalization.
- all new MS raw data files have been uploaded to PRIDE. Reviewers may use the following login credentials to access the data pending publication: username: reviewer_pxd069830@ebi.ac.uk, password: Z6N5ue4ToxNo.

We restructured the supplementary figures and tables in accordance with editorial guidelines as expanded view (EV) figures that are shown in the main text and additional figures and tables provided in an appendix. In addition, we have included detected mutant peptides in AP-LiP-MS data visualizations of kinase mutants (Figure 3A). All changes in the main text are highlighted in red.

All details are provided below.

Point-by-point response:

Reviewer #1:

The manuscript "Paradoxical non-catalytic kinase functions are driven by 1 inhibitor-induced displacement of autoinhibitory domains" by Reber et al. described an integrated mass spectrometry approach combining LiP-MS, AP-MS, and proximity labeling-MS to map the inhibitor-induced kinase conformation and interaction changes. This works is very interesting and uncover the widespread mechanisms of kinase inhibitor-induced non-catalytic conformation alterations, which might greatly contribute to the rational design of more effective kinase inhibitors. I think this work can be published on MSB after minor revision.

1. All the Lip-MS experiments were performed after affinity purification. Although this AP approach is more native than in vitro analysis, the sequence and site coverage is a problem. So, can the authors cover all the interesting regions of kinase in ATP binding and autoinhibitory domains? The sequence region coverages for all the protein kinase analysis should be given in the manuscript.

We more clearly indicated in our revised Fig. 1B, C and D, Fig. 3A, Fig. 5B, Fig. EV1C, Fig. EV2B and Fig. EV4E how the peptides identified by AP-LiP-MS cover the various annotated protein domains of the analyzed kinases and also added information on the % of sequence coverage. We also provide information on the domain level coverage in Appendix Tables S1, S3 and S5.

2. How the authors evaluate the kinase conformation changes is directly related to the inhibitor binding or by other protein interactions? As the AP-MS experiments identified many interaction proteins. Maybe some invitro analysis by using purified proteins is still needed?

We thank the reviewer for suggesting a more precise evaluation of the measured structural changes. In our view, the goal and advantage of AP-LiP-MS over other techniques is to measure structural changes induced by inhibitors in the context of native human kinases reflecting physiological PTMs and in association with kinase binding proteins. Purified recombinant proteins used for classical *in vitro* target engagement studies lack binding partners but also PTMs. This compromises their use to investigate relevant probe- induced structural changes. In fact, a recent study showed that ligandability can be modulated by PTMs[1]. We agree with the reviewer that the measured structural changes in response to inhibitors may be affected also by the presence/absence of these interactors (in addition to PTMs). We therefore included a sentence at page 4 line 124 in the first result section to clarify this point: "Since we analyze natively purified kinases, it is possible that some of these inhibitor-induced conformational changes may depend on the presence or absence of kinase binding partners or PTMs."

3. How the in vivo proximity labeling experiment and data analysis are conducted are not clear. They authors should describe the procedures in more detail. If CH2O is used for in vivo labeling, how to avoid crosslinking, how to do database searching and realize confident results?

We have better organized the corresponding subsections in the *Methods* section to make it clear how we conducted and analyzed our biotin proximity labeling experiments. CH2O was not used for *in vivo* labeling.

We used CH2O exclusively for chemical modification of the SrepTactin sepharose beads prior to the pulldown step to reduce the amount of streptavidin peptides following on bead digestion of *in vivo* proximity labeling samples based on a previously published protocol[2].

4. For AP-Lip-MS analysis, the conformation information of other proteins within the complexes could be also obtained. Can the authors also analysis and discuss these interface alterations.

We greatly appreciate this suggestion to identify structurally informative peptides of kinase interactors in our AP-LiP-MS analysis. Unfortunately, peptide coverage for kinase interactors was much lower compared to bait peptides (Fig. R1A), which limits the detection of structural changes by our method. For both CAMKK2 and PRKCA, none of the peptides from kinase interactors passed our statistical criteria for significance. However, AP-LiP MS identified significant changes in the CHEK1 interactor CLPB (Fig. R1B), which dissociated from CHEK1 upon treatment with rabusertib. We observed changes in a region with no functional annotation, in an ANK repeat which mediates protein-protein interactions, and in a segment that regulates disaggregase activity. This suggests that CLPB might interact with CHEK1 via these regions, and that CHEK inhibition leads to structural changes that could alter mitochondrial disaggregase activity of CLPB. This, in turn, could help to explain the observed changes in mitochondrial morphology upon CHEK1 inhibition. We have added the AP-LiP-MS data on CLPB in Fig. 5B and described this finding in the revised manuscript on page 9 line 275 as it supports our hypothesis that the change in the CHEK1-CLPB complex underlies the change in mitochondrial morphology.

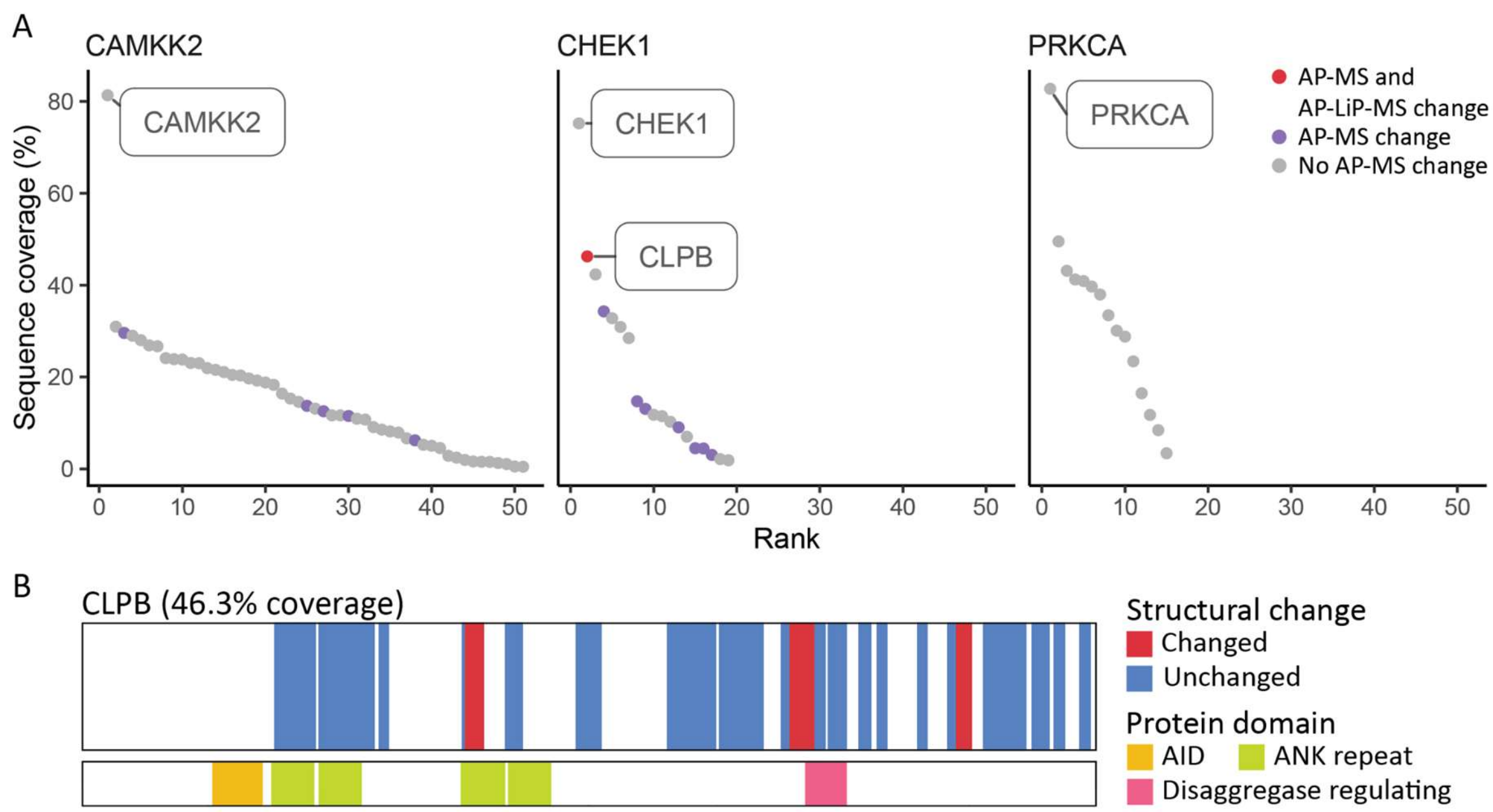

Figure R1. AP-LiP-MS changes in interactors. A) Comparison of sequence coverage for interactors of CAMKK2, CHEK1 and PRKCA. Proteins that show significant changes in AP-MS and AP-LiP-MS experiments are shown in red, those changing in AP-MS experiments only are shown in purple and all other interactors are shown in grey. B) Structural changes detected for CLPB upon CHEK1 inhibition with rabusertib. All AP-LiP-MS experiments were performed in triplicates and p-values were calculated using a moderated t-test followed by multiple testing corrections using the procedure of Benjamini-Hochberg.

Reviewer #2:

Summary

Reber et al. address an underexplored dimension of kinase pharmacology: the non-catalytic, "off-mechanism" effects of ATP-competitive inhibitors that is mediated through changes of kinase domain conformation and protein-protein interaction (PPI) changes. The authors develop a powerful proteomics framework using LiP-MS (limited proteolysis mass spectrometry; previously developed by the authors) applied to native afffinity-purified protein complexes, and proximity labeling-MS to systematically map inhibitor-induced structural and interactome remodeling in a native cellular context. Using three Ser/Thr-kinases (CAMKK2, CHEK1, and PRKCA) with distinct autoinhibitory domain (AID) architectures, Reber and colleagues demonstrate that ATP-competitive kinase inhibitor binding induces displacement of the AID, promoting an open, active-like conformation despite catalytic inhibition. This structural transition rewires PPIs and drives diverse (paradoxic) non-catalytic phenotypes. This is nicely demonstrated in relevant cell biological assays for all three kinase-inhibitor pairs. The data is supported by mutational analyses, which allows to disentangle catalytic inhibition from conformational effects. The findings support a model in which the ATP-binding site acts as a central allosteric hub governing kinase conformation and interaction networks.

Overall, the study provides compelling evidence that kinase inhibitor-induced neomorphic PPIs may be a widespread and understudied feature with potentially important biological functions. The proposed proteomics pipeline represents a scalable and generalizable strategy to uncover such effects, with important implications for drug development, particularly in anticipating side effects and improving inhibitor selectivity.

Major comments:

1. While I agree that kinase inhibitor-induced changes of (intra- and intermolecular) PPIs are understudied and some paradoxical effects of kinase inhibitor on signaling are mentioned in the Discussion section (paradoxical MAPK activation by BRAF inhibitors and JAK2 hyperphosphorylation by type I JAK1/2 inhibitors), one very prominent early example is missing and should be included: The Grzesiek lab demonstrated in 2013 that ATP competitive kinase inhibitors targeting BCR::ABL1 result in an open conformation, which enable its SH2 and SH3 domains to engage in PPIs (Skora et al. PNAS 2013; PMID: 24191057).

We thank the reviewer for pointing out the early example on the BCR:ABL1 inhibitor which we overlooked. We now included the work of Skora et al. in line 444 of the discussion of our revised manuscript on page 14 (see also response to question 2 below).

2. I was a bit puzzled that the authors focus entirely on type I kinase inhibitors. While type I inhibitors are indeed more common and induce an active open conformation of the kinase domain and are therefore prime candidates for the studied phenomenon, it has been shown that also type II inhibitors lead to AID displacement and disassembly of intramolecular PPIs. This was first demonstrated for type II BCR::ABL1

kinase inhibitors (see: Sonti et al. JACS 2018; PMID: 29319304); whereas in contrast type I TKIs (e.g. dasatinib, bosutinib, ...) kept BCR::ABL1 in an assembled conformation. I suggest to include these examples and thereby broaden the scope/impact of the manuscript. Hence, it can be expected that also binding of many type II inhibitors (and potentially also allosteric inhibitors) may also result in conformations that displace AID and induce neomorphic PPIs.

We thank the reviewer for noting this important point and agree that the described inhibitor responses may not be restricted to type I but also occur for type II inhibitors and potentially also allosteric inhibitors. We included the suggested examples and expanded on this point to broaden the scope of non catalytic effects in our discussion page 14, line 444: "While we and others have observed AID displacement and PPI rearrangements most commonly for type I inhibitors, there is evidence that also type II tyrosine kinase inhibitors (TKIs) can induce an open conformation. This was first demonstrated for the type II inhibitor imatinib against ABL kinase, which induced a disassembled state of the SH3-SH2-kinase core of ABL[3]. On the other hand, type I TKIs against ABL like dasatinib or bosutinib bound ABL in the assembled state[4]. This shows that structural modulation by kinase inhibitors is a phenomenon that also occurs for other types of inhibitors." We also highlight that also type II inhibitors can show similar effects in line 456.

3. The manuscript focuses on kinase inhibitor-induced changes on native (unmutated) kinases. While this is important to study for possible side effects in "off-tissues" or uncover diminished therapeutic effect in the on-target cells/tissues, I lack a discussion and mention that the therapeutic targets of most kinase inhibitors are not native kinases, but active oncogenic kinases that are activated by point mutations, insertions/deletions, duplication, fusion partners etc. These oncogenic kinase variants have often already displaced AIDs, open/disassembled conformations and possibly a different subcellular localization. What is the authors expectation of the consequences of inhibitor-induced (further) changes in this common scenario?

We thank the reviewer for suggesting a discussion on the importance of disease-associated target variants in profiling inhibitor responses. In our study we presented one example of a disease-associated target variant (CAMKK2 R311C) which indeed demonstrated that inhibitor induced PPI changes can depend on the disease mutant context. This is of course even more important for the analysis on how oncogenic target kinase variants are affected by inhibitors. We added text on page 14 line 462 in the discussion section to clarify this and to discuss the need to include most common disease associated target variants to better understand the inhibitors mechanism of action in the disease context: "Most of our analysis focused on inhibitor-induced changes of the corresponding WT kinase, which is particularly important to study both possible on-target side effects in healthy tissues or lack of efficacy in diseased tissues. The disease-associated CAMKK2 R311C mutant showed that responses to inhibitors may depend on the specific disease variant, which may affect their conformation, PPIs or subcellular localization[5]. Since most therapeutic kinase inhibitors aim to target kinases that are mutated in oncogenic contexts, it will be important to expand multimodal proteomic profiling also towards disease-associated target variants. In this regard…"

Reviewer #3:

The authors set out to investigate small molecule inhibitor functions beyond catalytic inhibition using a multi-proteomic approach. Focussing on three examples with different structural architecture and three matching selective small molecule inhibitors, structural re-arrangements via LIP-MS and the change of the proximal proteome are investigated upon ligand treatment. The authors identify three separate consequences of ligand binding beyond inhibition: for CHEK1 dissociation from CLBP is identified. This causes mitochondrial fragmentation. PRKCA is re-localised to cell junctions and endosome-like organelles. CAMKK2 triggers AMPK sequestration. The study is overall of very high quality, elucidates novel mechanisms of action beyond inhibition of inhibitors and thus provides timely insights into additional/off-target concequences of small molecule binding. This provides important insights into small molecule actions and further substantiates the need of detailed dissection of inhibitor consequences beyond inhibition. At this point a few open points still, however, remain unclear.

Major points:

1. The authors refer multiple times to neomorphic features. This would imply that a novel function of the protein is induced by small molecule binding. However, the authors do not specifically show any neomorphic features and instead it seems like endogenous interactions or functions are modulated by inhibitor binding?

We thank the reviewer for the suggestion to be more careful when using the term "neomorphic" since many of our reported inhibitor responses can indeed be explained by modulation of an already existing kinase function. To avoid confusion, we removed the term "neomorphic" in lines 76, 391 and 473 in the revised manuscript.

2. While the work is of high quality, all three inhibitors have IC50s in the nanomolar range, but are treated at 1 µM. This hampers the strength of the drawn conclusions. Are the observed effects of direct or indirect nature? Could the authors please provide evidence that ligand binding is directly causing the observed non-catalytic effects.

We thank the reviewer for acknowledging the quality of our work. The reviewer raises an important question that is relevant for all studies analyzing the effects of small molecule inhibitors. In order to detect inhibitor effects *in vivo*, we used concentrations higher than the known biochemical IC50 values to overcome known factors that reduce their *in vivo* capacity (millimolar intracellular ATP concentration, cellular uptake, pharmacokinetics). The 1 µM inhibitor concentration used in our study is well within the range of concentrations used in the literature for measuring *in vivo* responses[6-8]. We have also provided additional experimental evidence to support our claim that observed non-catalytic effects are direct consequences of ligand-target interactions.

To ensure specificity of the reported effects for CAMKK2, we used the corresponding negative control compound SGC-CAMKK2-1N, a compound that is structurally related to the active inhibitor SGC-CAMKK2-1 but does not bind CAMKK2[6]. In contrast to the active SGC-CAMKK2-1 inhibitor this molecule did not

cause changes in CAMKK2 structure or interaction with AMPK suggesting that the measured SGC-CAMKK2-1 effects depend on direct inhibitor binding (Fig R2). This new information is now included in the revised version in Fig. EV2 and described in the manuscript on page 5 line 153: "To ensure that the observed changes are specific, we used the negative control compound SGC-CAMKK2-1N[6] (Fig. EV2A). This compound is structurally similar to the inhibitor SGC-CAMKK2-1 but does not bind CAMKK2. We found that this compound does not induce any structural changes in CAMKK2 (Fig. EV2B, Appendix Fig. S4A, B) or alter its interactome (Fig. EV2C, Appendix Fig. S4C). Thus, the changes observed upon treatment with SGC-CAMKK2-1 are likely specific."

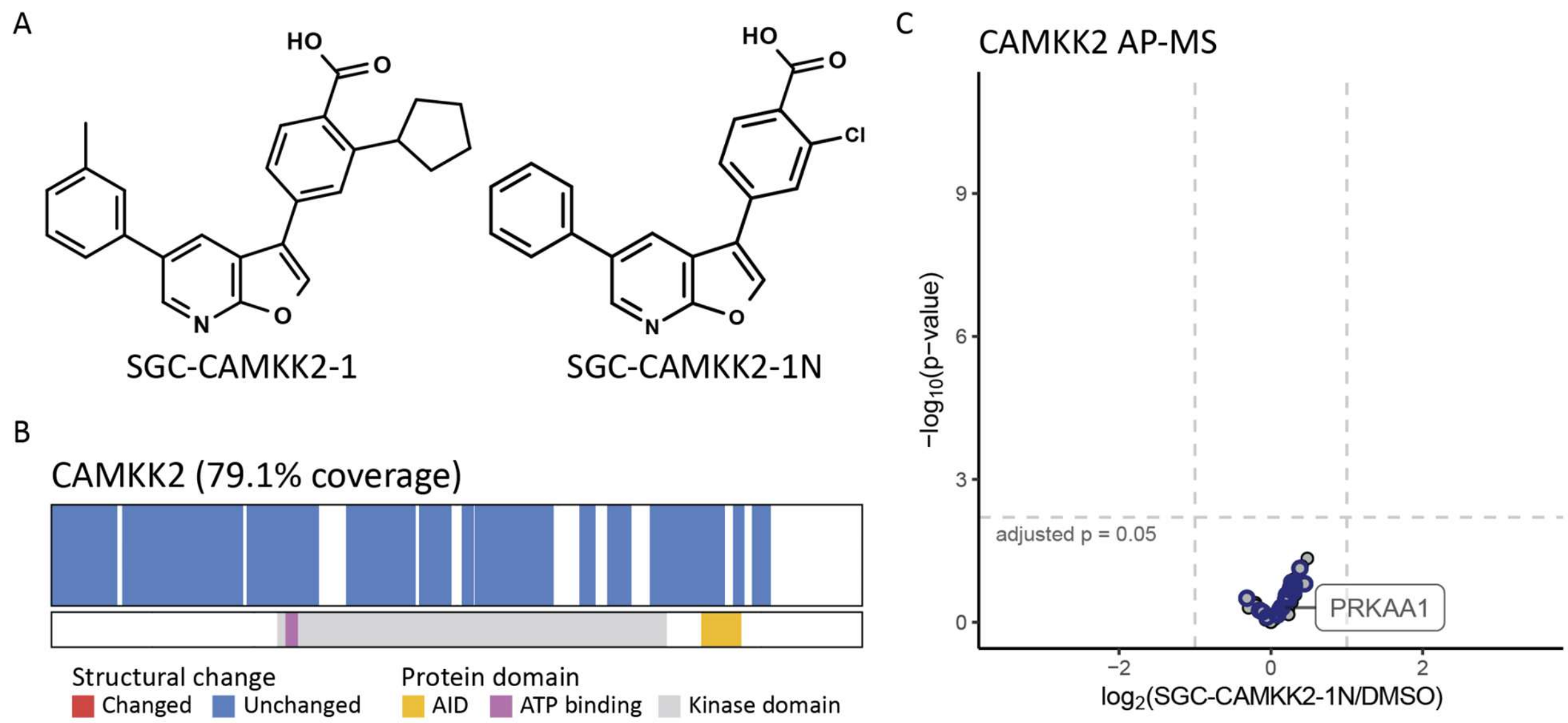

Figure R2. The negative control compound SGC-CAMKK2-1N does not induce structural or interaction changes in CAMKK2. A) Structures of the inhibitor SGC-CAMKK2-1 and the negative control compound SGC-CAMKK2-1N. B) Structural changes observed with AP-LiP-MS for CAMKK2 treated with SGC-CAMKK2-1N. C) Volcano plot illustrating changes in CAMKK2 interactors upon inhibition with SGC-CAMKK2-1N measured by AP-MS. Known CAMKK2 interactors are highlighted with a blue border. AP-LiP-MS experiments were performed in technical triplicates and AP-MS experiments were performed in biological triplicates. P-values were calculated using a moderated t-test followed by multiple testing corrections using the procedure of Benjamini-Hochberg.

Such a negative control compound is not available for the probes used to investigate CHEK1 and PRKCA, prompting us to apply other strategies.

The CHEK1 inhibitor rabusertib was ranked among the highest in selectivity among 243 kinase inhibitors tested in a chemoproteomics study with no off targets being detectable for rabusertib across 253 kinases even at high concentrations[9]. This does not fully exclude other indirect drug effects but demonstrates exceptional target selectivity. We reported that the CHEK1-CLPB complex dissociates after 4 h *in vivo* treatment with 1 µM rabusertib. By AP-LiP-MS, we observed structural changes in CLPB in CHEK1 pulldowns after only 5 minutes of rabusertib treatment of purified complexes *in vitro* (see Fig. R1B), suggesting that the observed CLPB dissociation from CHEK1 is direct. We also observed that the constitutively active CHEK1 L449R mutant no longer dissociates from CLPB, showing that the effect does

indeed depend on CHEK1 directly. Furthermore, in our manuscript we provided evidence that rabusertib-induced mitochondrial fragmentation cannot be explained by indirect effects such as DNA damage or CDK1 inactivation induced by rabusertib (Fig. EV3, Fig. 5).

For the PRKCA inhibitor Gö 6983, we addressed this question by measuring Ca^{2+} release upon Gö 6983 treatment and performing dose-response proximity labeling experiments. Since membrane association of PRKCA is regulated via Ca^{2+}-signaling[10], we already asked whether Gö 6983 may affect membrane relocalization indirectly via increasing intracellular Ca^{2+} levels However no Ca^{2+} increase could be detected upon inhibitor treatment which excludes such an indirect mechanism (Fig. EV4B). However, we acknowledge that this does not rule out non-specific effects via other mechanisms. We therefore performed an additional series of dose-response proximity labeling MS experiments following the well-established rationale that specific on target effects occur at lower concentrations than non-specific effects[11, 12]. We treated miniTurbo-PRKCA expressing cells with 6 different concentrations between 100 pM to 10 µM and plotted the response curves of all the proteins which changed their proximity to PRKCA across the concentration range (Fig. R3). The proteins we reported to change in Fig. 6A had a median half maximum effective concentration (EC50) of 382 nM, while all other proteins changed proximity only at much higher concentrations with a median EC50 of 2.1 µM. The difference between these groups is highly significant ($p = 1.8\times10^{-11}$, two-tailed Mann Whitney U test). This supports the view that the measured effects are highly specific.

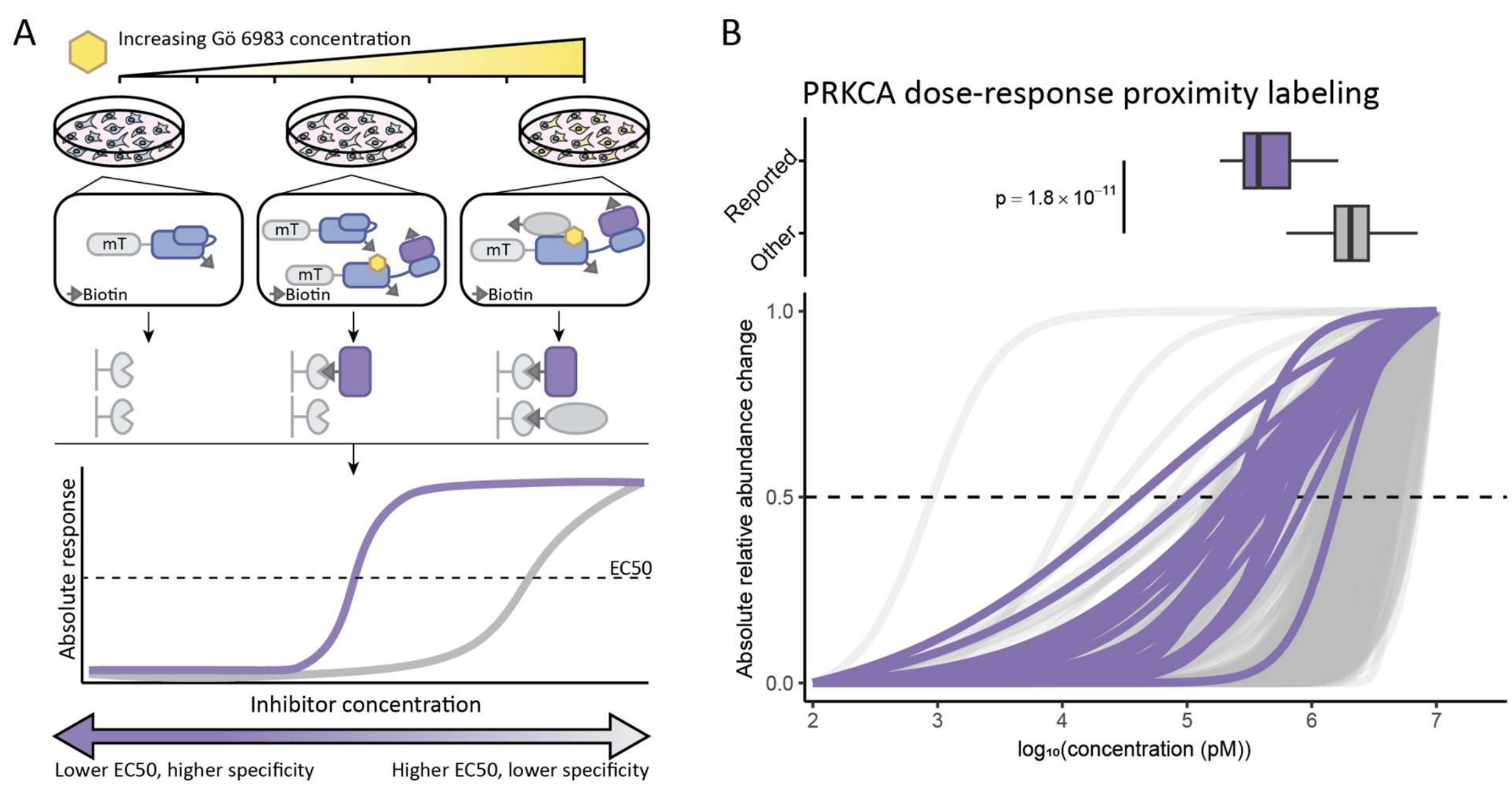

Figure R3. Dose-response *in vivo* proximity labeling shows that relocalization of PRKCA in response to Gö 6983 occurs at lower EC50s than other effects. A) Schematic representation of the dose-response *in vivo* proximity labeling workflow. B) Dose-response curves for proteins changing with increasing Gö 6983 concentrations. Proteins reported to change in Fig. 6A are shown in purple, all other proteins are shown in grey. For each dose, biological quadruplicates were used. The p-value comparing EC50s was calculated using a two-sided Mann Whitney U test.

These new results have been added to the manuscript in Fig. EV4C, D and are described starting at page 11, line 339: "To investigate whether the observed relocalization is specific, we followed previous approaches that have established that specific, on-target effects occur at lower concentrations compared to indirect off-target effects[11, 12]. We therefore performed a dose-response in vivo proximity labeling experiment (Fig. EV4C) with 6 treatment concentrations ranging from 100 pM to 10 µM and computed the half-maximal effective concentration (EC50) of all proteins for which we obtained a high-correlation and significantly changing fitted sigmoidal curve (Fig. EV4D, Appendix Fig. S9). We found that the changes reported in Fig. 6A occurred at significantly lower concentrations (median EC50 = 382 nM) compared to all other proteins (median EC50 = 2.1 µM; p = 1.8×10-11, two-tailed Mann Whitney U test)."

3. For PRKCA it would be interesting to see if mutated versions (inactive/constitutively active) occupy the same structural space to WT and how this correlates to the observed behavior upon ligand binding- if the hypothesis is correct Go6983 should lead to the identical structural changes.

We initially shared the view of the reviewer that the mutants should be structurally similar to the WT and thus undergo similar structural changes when treated with Gö 6983. In order to test this hypothesis, we performed a new set of AP-LiP MS experiments including the PRKCA variant D436A and R22A/A25E (Fig. R4A). While we had less overall protein coverage in the mutants, we noticed that the only peptides affected across all three experiments mapped to the C2 domain, which mediates PRKCA membrane binding[13]. The shared structural alterations in the C2 domain in WT and mutant PRKCA correlate well with the similar inhibitor induced proximity changes to membrane proteins. Together with the proposed role of the C2 domain in membrane targeting via direct interactions with phosphatidylinositol 4,5-bisphosphate[14], these results strengthen our model in which the inhibitor causes structural alterations in the C2 domain that drive membrane relocalization.

In contrast to PRKCA WT, Gö 6983 did not change the AID in the mutants. While this could be expected for the constitutively active R22A/A25E mutant[15], we did not expect this for D463A as we assumed it would have a closed conformation like PRKCA WT. To explore why we did not observe structural changes in the AID of the D463A mutant, we generated AlphaFold 3 models of the PRKCA KD-AID interaction following the same protocol as for CAMKK2-PRKAA1 (see *Methods*; Fig. R4B, C). These models suggest that D463 is located in the KD interface interacting with the AID. We believe that the D463A mutation might thus disrupt this domain-domain interaction, similar to the R22A/A25E mutant. This could explain why we do not see structural changes in the AID upon Gö 6983 treatment. Our AP-LiP-MS data together with the AlphaFold 3 prediction suggest that the D463A and R22A/A25E mutants do not occupy the same structural space as the WT but rather have unique conformations and thus undergo different structural changes upon Gö 6983 treatment. These new insights do not change our main conclusions about how Gö 6983 induces relocalization of PRKCA to the membrane via structural changes in the C2 domain. The new AP-LiP-MS results are now included in Fig. EV4E, Appendix Fig. S10, Appendix Table S5 and referenced on page 11 line 352 of the revised manuscript: "AP-LiP MS of PRKCA WT and mutants identified shared structural changes within the C2 domain when inhibited (Fig. EV4E, Appendix Fig. S10, Appendix Table S5) suggesting that membrane relocalization may be driven by structural alterations in the C2 domain."

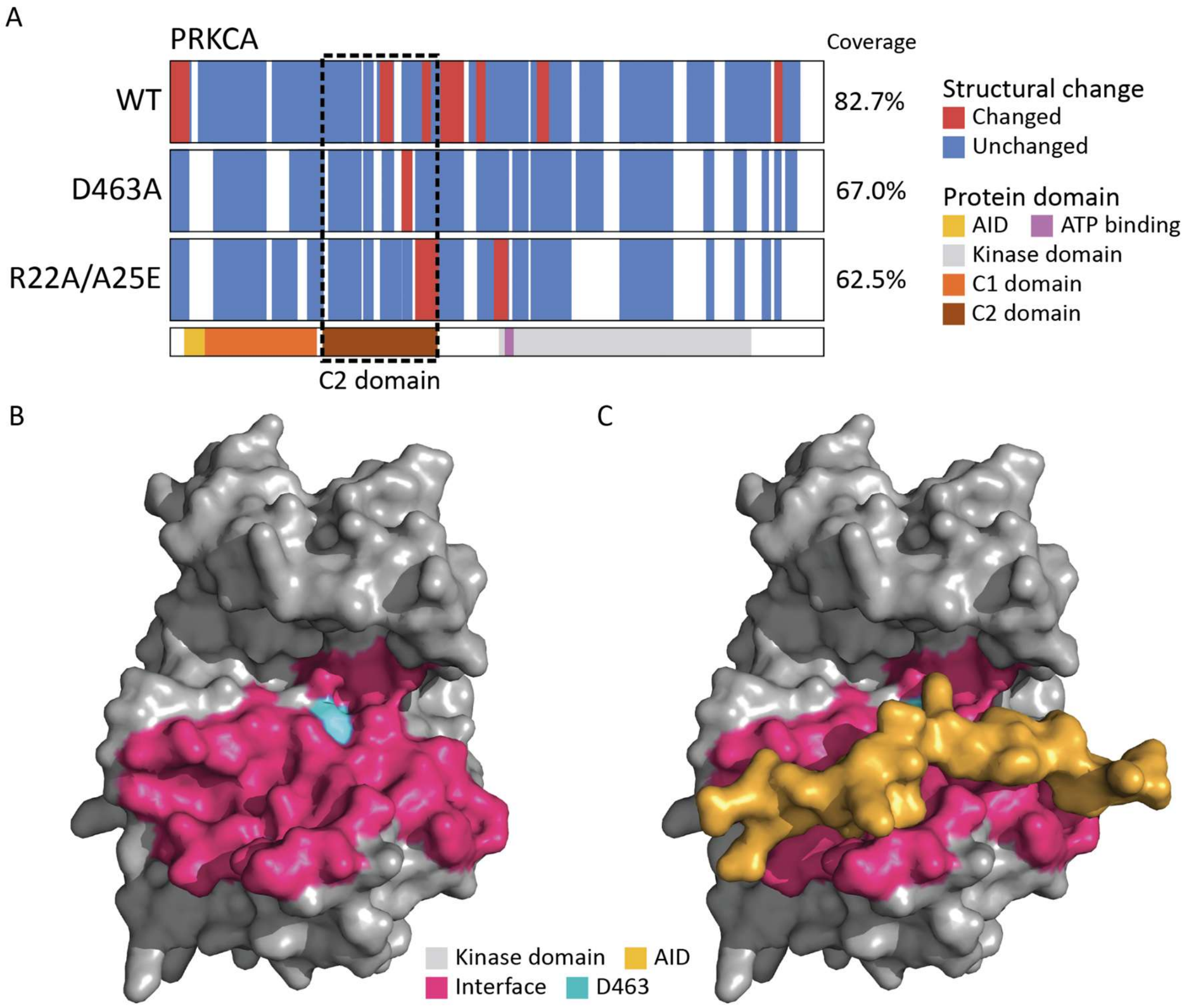

Figure R4. PRKCA mutants undergo structural changes in the C2 domain. A) Gö 6983 induced structural changes obtained by AP-LiP-MS in wild type and mutant PRKCA. The panel for PRKCA WT was previously shown in Fig. 1D. AP-LiP-MS experiments were performed in technical triplicates. P-values were calculated using a moderated t-test followed by multiple testing corrections using the procedure of Benjamini-Hochberg. B, C) AlphaFold 3 predictions of the PRKCA KD-AID interaction shown without (B) and with the AID (C). The catalytic residue D463 lies in the predicted interaction interface with the AID.

4. What percentage of loss of PRKCA in native lysis conditions upon Go6983 treatment can be explained by relocalisation to the cellular junctions vs the suggested insoluble organelles?

We were also curious about the distribution of PRKCA after Gö 6983 treatment. Using MS PRKCA abundance measurement in native lysates we estimate that 75% of PRKCA relocalizes into insoluble subcellular structures upon inhibitor treatment (Fig. EV4A). Super-resolution microscopy data of Gö 6983-treated HEK293 cells expressing EGFP-PRKCA was acquired as z-stacks as described in *Methods*. Deconvolved z-stacks were segmented in Ilastik 1[16] and signal intensity was integrated per cell over the volumes of the membranes and foci. Experiments were done in technical triplicates using 38 images per

cell. We estimate that 83.7% (SD = 8.0%) of PRKCA was present at junctional membranes and 16.2% (SD = 8.0%) at dot-like organelles in response to Gö 6983.

Minor points:

1. Line 17: The abstract lacks a definition of AIDs.

Thank you for noting the lacking abbreviation. We have added it to the abstract.

2. Line 24: The authors should consider rephrasing "highly prevalent" since here three examples are shown.

We have replaced “highly prevalent” with “likely to occur in other kinases as well” to tone down our statement.

3. Line 62: naming the inhibitors at this point may be beneficial, including reported EC50s or IC50s.

We added the name of the inhibitors and their known IC50s in line 60 of the revised manuscript.

4. Line 111: should include details as to binding affinity/inhibitory capacity and selectivity (see also comment on line 62).

We added more details on available half maximum inhibitory capacity in line 60 and references to available selectivity data in line 63.

5. Line 361: it is unclear in what context "more pervasive feature" is meant.

We agree the term is not clear in this context and we replaced it with “could be a common feature” in line 383 of the revised manuscript.

References:

1. Li, W., Wei, Q., Llanos, M., Gathmann, C., Governa, P., Chiu, T.Y., Wozniak, J.M., Jadhav, A.M., Holcomb, M., Cravatt, J., Dongre, A. et al. Posttranslational modifications remodel proteome-wide ligandability. *Nat Chem Biol* (2026).
2. Rafiee, M.R., Sigismondo, G., Kalxdorf, M., Forster, L., Brugger, B., Bethune, J. & Krijgsveld, J. Protease-resistant streptavidin for interaction proteomics. *Mol Syst Biol* **16**, e9370 (2020).
3. Skora, L., Mestan, J., Fabbro, D., Jahnke, W. & Grzesiek, S. NMR reveals the allosteric opening and closing of Abelson tyrosine kinase by ATP-site and myristoyl pocket inhibitors. *Proc Natl Acad Sci USA* **110**, E4437–E4445 (2013).
4. Sonti, R., Hertel-Hering, I., Lamontanara, A.J., Hantschel, O. & Grzesiek, S. ATP Site Ligands Determine the Assembly State of the Abelson Kinase Regulatory Core via the Activation Loop Conformation. *J Am Chem Soc* **140**, 1863–1869 (2018).
5. Lacoste, J., Haghighi, M., Haider, S., Reno, C., Lin, Z.Y., Segal, D., Qian, W.W., Xiong, X., Teelucksingh, T., Miglietta, E., Shafqat-Abbasi, H. et al. Pervasive mislocalization of pathogenic coding variants underlying human disorders. *Cell* **187**, 6725–6741 e6713 (2024).

6. Wells, C., Liang, Y., Pulliam, T.L., Lin, C., Awad, D., Eduful, B., O'Byrne, S., Hossain, M.A., Catta-Preta, C.M.C., Ramos, P.Z., Gileadi, O. et al. SGC-CAMKK2-1: A Chemical Probe for CAMKK2. *Cells* **12**, 287 (2023).
7. Koppenhafer, S.L., Goss, K.L., Terry, W.W. & Gordon, D.J. Inhibition of the ATR-CHK1 Pathway in Ewing Sarcoma Cells Causes DNA Damage and Apoptosis via the CDK2-Mediated Degradation of RRM2. *Mol Cancer Res* **18**, 91–104 (2020).
8. Violin, J.D., Zhang, J., Tsien, R.Y. & Newton, A.C. A genetically encoded fluorescent reporter reveals oscillatory phosphorylation by protein kinase C. *J Cell Biol* **161**, 899–909 (2003).
9. Klaeger, S., Heinzlmeir, S., Wilhelm, M., Polzer, H., Vick, B., Koenig, P.A., Reinecke, M., Ruprecht, B., Petzoldt, S., Meng, C., Zecha, J. et al. The target landscape of clinical kinase drugs. *Science* **358** (2017).
10. Medkova, M. & Cho, W. Mutagenesis of the C2 domain of protein kinase C-alpha. Differential roles of Ca2+ ligands and membrane binding residues. *J Biol Chem* **273**, 17544–17552 (1998).
11. Piazza, I., Beaton, N., Bruderer, R., Knobloch, T., Barbisan, C., Chandat, L., Sudau, A., Siepe, I., Rinner, O., de Souza, N., Picotti, P. et al. A machine learning-based chemoproteomic approach to identify drug targets and binding sites in complex proteomes. *Nat Commun* **11**, 4200 (2020).
12. Bayer, F.P., Gander, M., Kuster, B. & The, M. CurveCurator: a recalibrated F-statistic to assess, classify, and explore significance of dose-response curves. *Nat Commun* **14**, 7902 (2023).
13. Evans, J.H., Murray, D., Leslie, C.C. & Falke, J.J. Specific translocation of protein kinase Calpha to the plasma membrane requires both Ca2+ and PIP2 recognition by its C2 domain. *Mol Biol Cell* **17**, 56–66 (2006).
14. Sanchez-Bautista, S., Marin-Vicente, C., Gomez-Fernandez, J.C. & Corbalan-Garcia, S. The C2 domain of PKCalpha is a Ca2+ -dependent PtdIns(4,5)P2 sensing domain: a new insight into an old pathway. *J Mol Biol* **362**, 901–914 (2006).
15. Ueda, Y., Hirai, S., Osada, S., Suzuki, A., Mizuno, K. & Ohno, S. Protein kinase C activates the MEK-ERK pathway in a manner independent of Ras and dependent on Raf. *J Biol Chem* **271**, 23512–23519 (1996).
16. Berg, S., Kutra, D., Kroeger, T., Straehle, C.N., Kausler, B.X., Haubold, C., Schiegg, M., Ales, J., Beier, T., Rudy, M., Eren, K. et al. ilastik: interactive machine learning for (bio)image analysis. *Nat Methods* **16**, 1226–1232 (2019).

15th Jun 2026

Manuscript Number: MSB-2026-13670R
Title: Paradoxical non-catalytic kinase functions are driven by inhibitor-induced displacement of autoinhibitory domains
Author: Viviane Reber
Sabrina Keller
Stefanie Loosli
Yumi Arima
Tatjana Kleele
Paola Picotti
Matthias Gstaiger

Dear Dr. Gstaiger,

Thank you for sending us your revised manuscript. We have now heard back from the reviewer who was asked to re-evaluate your study. As you will see below, the reviewer is now satisfied with the modifications made and supports publication. Before we can formally accept your manuscript, we would ask you to address the following editorial-level issues:

1. Please remove the figures from the manuscript file, and the figure legends should be placed after the References section.

2. The submission system lists two corresponding authors, whereas only one corresponding author is indicated in the manuscript file. This discrepancy should be resolved.

3. "Disclosure statement and competing interests" should be renamed to "Disclosure and Competing Interests Statement".

4. The references need to be formatted according to the Molecular Systems Biology reference style. Please list up to 10 co-authors of a paper before adding et al. in the reference list. Citations should be listed in alphabetical order.

5. Appendix: The title page should include "Appendix for [Manuscript Title]" (author names and affiliations are not required) and a Table of Contents listing all appendix items with corresponding page numbers. Figures and tables should be consistently labeled as Appendix Figure Sx and Appendix Table Sx throughout both the manuscript file and the Appendix PDF. The term "Supplementary" should not be used.

6. Data availability:Please ensure that all the datasets will be made publicly available upon the acceptance of the manuscript.

7. Please provide a completed Source Data Checklist and ensure that all information entered in the checklist is consistent with the source data files provided.

8. Please note that the box plots need to be defined in terms of minima, maxima, centre, bounds of box and whiskers, and percentile in the legends of figures EV3 B, EV4D

9. Sections need to be named and the order should be corrected to: Title page - Abstract - Introduction - Results - Discussion - Methods - Data Availability - Acknowledgements - Disclosure and Competing Interests Statement - References - Figure Legends - Table(s) - Expanded View Figure Legends.

Use the following link to submit your revised paper:

https://msb.msubmit.net/cgi-bin/main.plex

As a matter of course, please make sure that you have correctly followed the instructions for authors as given on the submission website.

Thank you for submitting this interesting paper to Molecular Systems Biology.

Kind regards,
Jingyi

Jingyi Hou, PhD

Senior Editor
Molecular Systems Biology

*** PLEASE NOTE *** As part of the EMBO Press transparent editorial process initiative (see our Editorial at https://dx.doi.org/10.1038/msb.2010.72 , Molecular Systems Biology will publish online a Review Process File to accompany accepted manuscripts. When preparing your letter of response, please be aware that in the event of acceptance, your cover letter/point-by-point document will be included as part of this File, which will be available to the scientific community. More information about this initiative is available in our Instructions to Authors. If you have any questions about this initiative, please contact the editorial office (msb@embo.org).

Reviewer #3:

The authors have addressed all raised points with very detailed experiments, including orthogonal approaches when chemical probes were not available. The manuscript points to features of kinase inhibitors beyond inhibition, which are important to highlight for the research community. I would hence recommend its acceptance for publication.

All editorial and formatting issues were resolved by the authors.

18th Jun 2026
Manuscript number: MSB-2026-13670RR
Title: Paradoxical non-catalytic kinase functions are driven by inhibitor-induced displacement of autoinhibitory domains

Dear Dr. Gstaiger,

Thank you again for sending us your revised manuscript. We are now satisfied with the modifications made and I am pleased to inform you that your paper has been accepted for publication.

Your manuscript will be processed for publication by EMBO Press. It will be copy edited and you will receive page proofs prior to publication. Please note that you will be contacted by Springer Nature Author Services to complete licensing and payment information.

You may qualify for financial assistance for your publication charges - either via a Springer Nature fully open access agreement or an EMBO initiative. Check your eligibility: https://link.springer.com/journal/44320/how-to-publish-with-us

Should you be planning a Press Release on your article, please notify the production team via this form in order to coordinate publication and release dates: https://forms.cloud.microsoft/e/eH4A8JVP6g

If you have any questions, please do not hesitate to contact the Editorial Office. Thank you for your contribution to Molecular Systems Biology.

Kind regards,
Jingyi

Jingyi Hou, PhD
Senior Editor
Molecular Systems Biology

>>> Please note that it is Molecular Systems Biology policy for the transcript of the editorial process (containing referee reports and your response letter) to be published as an online supplement to each paper. If you do NOT want this, you will need to inform the Editorial Office via email immediately. More information is available here: https://link.springer.com/partners/embo-press/editorial-policies#Peer%20review

EMBO Press Author Checklist

Corresponding Author Name: Matthias Gstaiger
Journal Submitted to: Molecular Systems Biology
Manuscript Number: MSB-2026-13670

USEFUL LINKS FOR COMPLETING THIS FORM
The EMBO Journal - Author Guidelines
EMBO Reports - Author Guidelines
Molecular Systems Biology - Author Guidelines
EMBO Molecular Medicine - Author Guidelines

Reporting Checklist for Life Science Articles (updated January
This checklist is adapted from Materials Design Analysis Reporting (MDAR) Checklist for Authors. MDAR establishes a minimum set of requirements in transparent reporting in the life sciences (see Statement of Task: 10.31222/osf.io/9sm4x). Please follow the journal's guidelines in preparing your
Please note that a copy of this checklist will be published alongside your article.

Abridged guidelines for figures
1. Data
The data shown in figures should satisfy the following conditions:
- the data were obtained and processed according to the field's best practice and are presented to reflect the results of the experiments in an accurate and unbiased manner.
- ideally, figure panels should include only measurements that are directly comparable to each other and obtained with the same assay.
- plots include clearly labeled error bars for independent experiments and sample sizes. Unless justified, error bars should not be shown for technical
- if n<5, the individual data points from each experiment should be plotted. Any statistical test employed should be justified.
- Source Data should be included to report the data underlying figures according to the guidelines set out in the authorship guidelines on Data

2. Captions
Each figure caption should contain the following information, for each panel where they are relevant:
- a specification of the experimental system investigated (eg cell line, species name).
- the assay(s) and method(s) used to carry out the reported observations and measurements.
- an explicit mention of the biological and chemical entity(ies) that are being measured.
- an explicit mention of the biological and chemical entity(ies) that are altered/varied/perturbed in a controlled manner.
- the exact sample size (n) for each experimental group/condition, given as a number, not a range;
- a description of the sample collection allowing the reader to understand whether the samples represent technical or biological replicates (including how many animals, litters, cultures, etc.).
- a statement of how many times the experiment shown was independently replicated in the laboratory.
- definitions of statistical methods and measures:
  - common tests, such as t-test (please specify whether paired vs. unpaired), simple χ2 tests, Wilcoxon and Mann-Whitney tests, can be unambiguously identified by name only, but more complex techniques should be described in the methods section;
  - are tests one-sided or two-sided?
  - are there adjustments for multiple comparisons?
  - exact statistical test results, e.g., P values = x but not P values < x;
  - definition of 'center values' as median or average;
  - definition of error bars as s.d. or s.e.m.

Please complete ALL of the questions below.
Select "Not Applicable" only when the requested information is not relevant for your study.

Materials

Newly Created Materials	Information included in the manuscript?	In which section is the information available? (Reagents and Tools Table, Materials and Methods, Figures, Data Availability Section)
New materials and reagents need to be available; do any restrictions apply?	Yes	Materials and Methods, Appendix

Antibodies	Information included in the manuscript?	In which section is the information available? (Reagents and Tools Table, Materials and Methods, Figures, Data Availability Section)
For **antibodies** provide the following information:<br>- Commercial antibodies: RRID (if possible) or supplier name, catalogue number and or/clone number<br>- Non-commercial: RRID or citation	Yes	Materials and Methods

DNA and RNA sequences	Information included in the manuscript?	In which section is the information available? (Reagents and Tools Table, Materials and Methods, Figures, Data Availability Section)
**Short novel DNA or RNA including primers, probes**: provide the sequences.	Yes	Appendix

Cell materials	Information included in the manuscript?	In which section is the information available? (Reagents and Tools Table, Materials and Methods, Figures, Data Availability Section)
**Cell lines:** Provide species information, strain. Provide accession number in repository **OR** supplier name, catalog number, clone number, and/**OR** RRID.	Yes	Materials and Methods, Appendix
**Primary cultures:** Provide species, strain, sex of origin, genetic modification status.	Not Applicable	
Report if the cell lines were recently **authenticated** (e.g., by STR profiling) and tested for mycoplasma contamination.	Yes	Materials and Methods

Experimental animals	Information included in the manuscript?	In which section is the information available? (Reagents and Tools Table, Materials and Methods, Figures, Data Availability Section)
**Laboratory animals or Model organisms:** Provide species, strain, sex, age, genetic modification status. Provide accession number in repository **OR** supplier name, catalog number, clone number, **OR** RRID.	Not Applicable	
**Animal observed in or captured from the field:** Provide species, sex, and age where possible.	Not Applicable	
Please detail **housing and husbandry conditions**.	Not Applicable	

Plants and microbes	Information included in the manuscript?	In which section is the information available? (Reagents and Tools Table, Materials and Methods, Figures, Data Availability Section)
**Plants:** provide species and strain, ecotype and cultivar where relevant, unique accession number if available, and source (including location for collected wild specimens).	Not Applicable	
**Microbes:** provide species and strain, unique accession number if available, and source.	Yes	Materials and Methods

Human research participants	Information included in the manuscript?	In which section is the information available? (Reagents and Tools Table, Materials and Methods, Figures, Data Availability Section)
If collected and within the bounds of privacy constraints report on age, sex and gender or ethnicity for all study participants.	Not Applicable	

Core facilities	Information included in the manuscript?	In which section is the information available? (Reagents and Tools Table, Materials and Methods, Figures, Data Availability Section)
If your work benefited from core facilities, was their service mentioned in the acknowledgments section?	Yes	Acknowledgements

Design

Study protocol	Information included in the manuscript?	In which section is the information available? (Reagents and Tools Table, Materials and Methods, Figures, Data Availability Section)
If study protocol has been **pre-registered, provide DOI in the manuscript**. For clinical trials, provide the trial registration number **OR** cite DOI.	Not Applicable	
Report the **clinical trial registration number** (at ClinicalTrials.gov or equivalent), where applicable.	Not Applicable	

Laboratory protocol	Information included in the manuscript?	In which section is the information available? (Reagents and Tools Table, Materials and Methods, Figures, Data Availability Section)
Provide DOI OR other citation details if **external detailed step-by-step protocols** are available.	Yes	Materials and Methods

Experimental study design and statistics	Information included in the manuscript?	In which section is the information available? (Reagents and Tools Table, Materials and Methods, Figures, Data Availability Section)
Include a statement about **sample size** estimate even if no statistical methods were used.	Yes	
Were any steps taken to minimize the effects of subjective bias when allocating animals/samples to treatment (e.g. **randomization procedure**)? If yes, have they been described?	Not Applicable	
Include a statement about **blinding** even if no blinding was done.	Not Applicable	
Describe **inclusion/exclusion criteria** if samples or animals were excluded from the analysis. Were the criteria pre-established?<br><br>If sample or data points were omitted from analysis, report if this was due to attrition or intentional exclusion and provide justification.	Not Applicable	
For every figure, are **statistical tests** justified as appropriate? Do the data meet the assumptions of the tests (e.g., normal distribution)? Describe any methods used to assess it. Is there an estimate of variation within each group of data? Is the variance similar between the groups that are being statistically compared?	Yes	Figure legends, Results, Materials and Methods

Sample definition and in-laboratory replication	Information included in the manuscript?	In which section is the information available? (Reagents and Tools Table, Materials and Methods, Figures, Data Availability Section)
In the figure legends: state number of times the experiment was **replicated** in laboratory.	Yes	Figure legends
In the figure legends: define whether data describe **technical or biological replicates**.	Yes	Figure legends

Ethics

Ethics	Information included in the manuscript?	In which section is the information available? (Reagents and Tools Table, Materials and Methods, Figures, Data Availability Section)
Studies involving **human participants**: State details of **authority granting ethics approval** (IRB or equivalent committee(s), provide reference number for approval.	Not Applicable	
Studies involving **human participants**: Include a statement confirming that **informed consent** was obtained from all subjects and that the experiments conformed to the principles set out in the WMA Declaration of Helsinki and the Department of Health and Human Services Belmont Report.	Not Applicable	
Studies involving **human participants:** For publication of **patient photos**, include a statement confirming that consent to publish was obtained.	Not Applicable	
Studies involving experimental **animals**: State details of **authority granting ethics approval** (IRB or equivalent committee(s), provide reference number for approval. Include a statement of compliance with ethical regulations.	Not Applicable	
Studies involving **specimen and field samples:** State if relevant **permits** obtained, provide details of authority approving study; if none were required, explain why.	Not Applicable	

Dual Use Research of Concern (DURC)	Information included in the manuscript?	In which section is the information available? (Reagents and Tools Table, Materials and Methods, Figures, Data Availability Section)
Could your study fall under dual use research restrictions? Please check biosecurity documents and list of **select agents and toxins** (CDC): https://www.selectagents.gov/sat/list.htm	Not Applicable	
If you used a select agent, is the security level of the lab appropriate and reported in the manuscript?	Not Applicable	
If a study is subject to dual use research of concern regulations, is the name of the **authority granting approval and reference number** for the regulatory approval provided in the manuscript?	Not Applicable	

Reporting

The MDAR framework recommends adoption of discipline-specific guidelines, established and endorsed through community initiatives. Journals have their own policy about requiring specific guidelines and recommendations to complement MDAR.

Adherence to community standards	Information included in the manuscript?	In which section is the information available? (Reagents and Tools Table, Materials and Methods, Figures, Data Availability Section)
State if relevant guidelines or checklists (e.g., **ICMJE, MIBBI, ARRIVE, PRISMA**) have been followed or provided.	Not Applicable	
For **tumor marker prognostic studies,** we recommend that you follow the **REMARK** reporting guidelines (see link list at top right). See author guidelines, under 'Reporting Guidelines'. Please confirm you have followed these guidelines.	Not Applicable	
For **phase II and III randomized controlled trials**, please refer to the **CONSORT** flow diagram (see link list at top right) and submit the CONSORT checklist (see link list at top right) with your submission. See author guidelines, under 'Reporting Guidelines'. Please confirm you have submitted this list.	Not Applicable	

Data Availability

Data availability	Information included in the manuscript?	In which section is the information available? (Reagents and Tools Table, Materials and Methods, Figures, Data Availability Section)
Have **primary datasets** been deposited according to the journal's guidelines (see 'Data Deposition' section) and the respective accession numbers provided in the Data Availability Section?	Yes	Data availability
Were **human clinical and genomic datasets** deposited in a public access-controlled repository in accordance to ethical obligations to the patients and to the applicable consent agreement?	Not Applicable	
Are **computational models** that are central and integral to a study available without restrictions in a machine-readable form? Were the relevant accession numbers or links provided?	Not Applicable	
If publicly available data were reused, provide the respective **data citations in the reference list**.	Yes	Materials and Methods, Data availability
